# The Malleability of Developmental Trends in Neutral and Negative Memory Illusions

**DOI:** 10.1037/xge0000127

**Published:** 2016-01

**Authors:** Henry Otgaar, Mark L. Howe, Nathalie Brackmann, Tom Smeets

**Affiliations:** 1 Faculty of Psychology and Neuroscience, Maastricht University and Department of Psychology, City University London; 2 Faculty of Psychology and Neuroscience, Maastricht University

**Keywords:** false memory, memory development, suggestion, misinformation, developmental reversal

## Abstract

Among many legal professionals and memory researchers there exists the assumption that susceptibility to false memory decreases with age. In 4 misinformation experiments, we show that under conditions that focus on the meaning of experiences, children are not always the most susceptible to suggestion-induced false memories. We begin by presenting a short overview of previous developmental false memory studies, the majority of which have found that the susceptibility to misinformation decreases with age. In Experiment 1, 6/7-year-olds, 11/12-year-olds, and adults received a video and were confronted with misinformation about related but nonpresented details. Older children and adults had higher misinformation acceptance rates than younger children. In Experiment 2, we replicated this finding adding a younger child group (4/6-year-olds). In Experiments 3 and 4, we used new material and again found that susceptibility to misinformation increased with age. Together, these experiments show that children’s memory accuracy is not necessarily inferior to that of adults.’

In most child development textbooks, readers find that older children typically outperform younger children on most if not all tasks. This includes measures of learning, memory, reasoning, and complex problem solving. However, recently there have been a number of counterintuitive developmental findings reported in the literature, ones that show that younger children outperform older children. For example, sometimes, younger children generate more creative problem solutions than older children (e.g., generate more alternative ways to use tools; see Defeyter & German, 2003) or find it easier to learn certain unusual abstract causal principals (see Gopnik, Griffiths, & Lucas, 2015). In memory development, younger children can sometimes be *less* susceptible to memory illusions than older children and adults (Brainerd, Reyna, & Ceci, 2008). Under these conditions, younger children can be *better* eyewitnesses than older children or adults.

These so-called *developmental reversals* mark an important exception to the standard textbook aphorisms. However, there is at least one developmental aphorism that has remained despite these recently reported reversals. Specifically, false memories induced by *suggestion* tend to decline gradually between childhood and adulthood (Brainerd, 2013; Bruck & Ceci, 1999; Ceci & Bruck, 1993). This developmental trend has been the focus of considerable research, not only because it is of theoretical relevance when trying to understand memory changes that occur with age, but also because it has important forensic implications. Specifically, the law is concerned with how children’s susceptibility to memory illusions makes them less reliable witnesses in judicial proceedings. Indeed, oftentimes, legal professionals regard children’s evidentiary statements as being less credible than adults’ (e.g., Bruer & Pozzulo, 2014a; Knutsson & Allwood, 2014) because of children’s greater susceptibility to suggestion-induced memory illusions.

The assumption that children are more susceptible to memory illusions than adults is also shared among many memory researchers. For example, Sutherland and Hayne (2001, p. 388) observed that, “the general finding is that suggestibility decreases as a function of age.” Recently, McGuire, London, and Wright (2015, p. 334) voiced a similar conclusion that “previous suggestibility and misinformation studies [has indicated] that false memory declines with age.” Such statements reinforce the long-held belief that as children mature, suggestion- and misinformation-based false memory rates decrease.

Over the past few decades, research has accumulated that shows that another type of memory illusion, *spontaneous* false memories, exhibits the opposite developmental pattern. Spontaneous false memories are ones that arise without any external pressure and occur as a consequence of the activation of related information in an individual’s knowledge base. Research has demonstrated that these false memories increase significantly between childhood and adulthood, a phenomenon that has been dubbed *developmental reversal* (Brainerd, 2013). In the current studies, we assessed whether the common developmental trend in suggestion-based memory illusions can also be “reversed” such that younger children are less susceptible to suggestion than older children and adults. These experiments emerged from our speculation that the standard developmental trend in suggestion-induced false memories can be attenuated or even reversed when applying theoretical principles borrowed from research on the development of spontaneous false memories. Before presenting these experiments, we provide a brief overview of developmental trajectories in both spontaneous and suggestion-induced false memories.

## Developmental Trends and False Memory Paradigms

Several paradigms include *suggestive pressure* in order to create false memories. An often-used method is the misinformation paradigm (Loftus, 2005), a procedure that has three stages: Participants first witness an event (e.g., see a video of an unarmed theft), are then presented with misinformation about the event (e.g., they are told that the culprit carried a gun), and finally, participants receive a memory test. What studies have shown is that about 30% of participants falsely remember seeing the suggested detail (e.g., the gun) in the original event. This result is known as the misinformation effect and it is more pronounced in younger than older children and adults (Loftus, 2011; Otgaar, Candel, Smeets, & Merckelbach, 2010).

Importantly, suggestive pressure and misinformation are not the same. Misinformation usually involves the subsequent presentation of (related) information that was not part of the original event. Suggestive pressure can occur in a number of different forms, ranging from the suggestion that an event occurred in a person’s life when in fact it never did (memory implantation paradigm; e.g., see Otgaar et al., 2012) to more subtle forms in which false memories are created when eyewitnesses talk to each other and include false details about what was witnessed (memory conformity paradigm; e.g., see Wright, Memon, Skagerberg, & Gabbert, 2009).

The most frequent method used to induce *spontaneous* false memories is the Deese/Roediger-McDermott (DRM) paradigm (Deese, 1959; Roediger & McDermott, 1995). Here, participants are presented with word lists containing associatively related words (e.g., *tears, sorrow, baby*). The meanings of these words converge on a nonpresented word known as the critical lure (i.e., *cry*). A robust finding is that the critical lure is often erroneously remembered during recall or recognition tests (Brainerd et al., 2008). Of relevance for the current studies is that these spontaneous false memories increase with age in childhood, a finding that has intrigued researchers because the trend is the opposite of the developmental decreases associated with suggestion-based memory illusions (Brainerd et al., 2008; Brainerd, Reyna, & Forrest, 2002; Howe, Wimmer, Gagnon, & Plumpton, 2009; Otgaar, Howe, Peters, Sauerland, & Raymaekers, 2013). This developmental reversal has not only been detected when using DRM lists but also with other meaning-connected procedures such as when categorized materials or pictures are presented (e.g., Bruer & Pozzulo, 2014b; Howe, 2006, 2008; Sloutsky & Fisher, 2004). When considered together, these findings are somewhat perplexing because they suggest that young children are both more and less susceptible to memory illusions than older children and adults.

**Figure 1 fig1:**
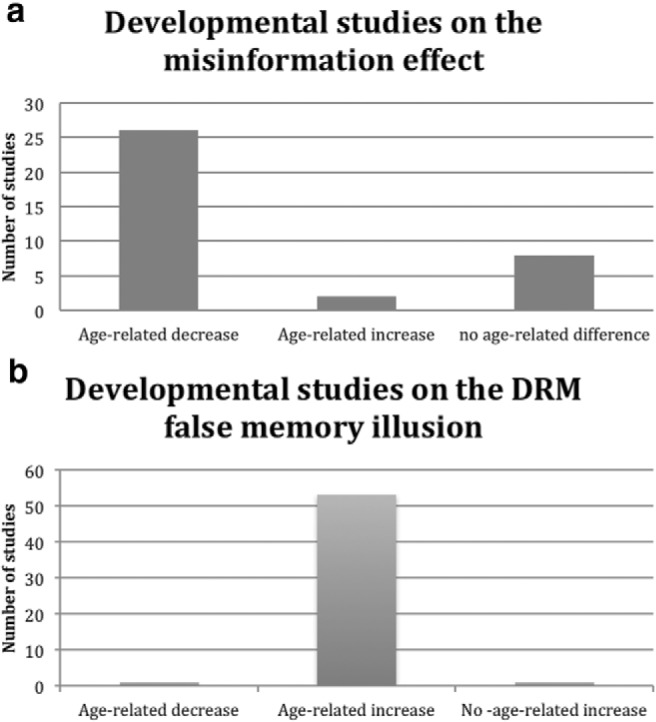
Developmental studies on the misinformation effect (1a) and the DRM false memory illusion (1b). 1a Note: Studies that had different developmental results for different conditions were included several times in the graph. 1b Note: These data have been reported in Brainerd et al. (2011). DRM = Deese/Roediger-McDermott.

To illustrate these different developmental patterns, we first examined the available literature on age-related trends in suggestion-induced false memories using the misinformation paradigm.^1^ We came across 29 articles in which findings were described about the development of false memories using the classic misinformation paradigm (see Table 1). As we anticipated, Figure 1a shows that the majority of these papers have found an age-related decrease in susceptibility to misinformation, a pattern that confirms ours as well as others’ (e.g., McGuire et al., 2015) view about typical developmental trends in suggestion-induced false memories. This contrasts nicely with Figure 1b that shows the exact opposite developmental pattern for spontaneous false memories using the DRM paradigm (taken from Brainerd, Reyna, & Zember, 2011).[Table-anchor tbl1][Fig-anchor fig1]

## Explaining Developmental Reversals in Spontaneous False Memories

This developmental reversal effect is anticipated by a number of theories. For example, Fuzzy-Trace Theory (FTT; Brainerd et al., 2008) assumes that experiences are stored using two opposing memory traces, verbatim and gist. Verbatim traces are involved in the storage of item-specific, detailed surface characteristics of an event whereas gist traces are related to the underlying meaning of an event. In FTT, false memories occur when verbatim traces are not available at retrieval and people rely on gist traces. Retrieved information that is consistent with the underlying meaning of an experience can be falsely recollected in the absence of contradictory verbatim information. Because the ability to extract the gist of experiences increases with age, and because it is not always possible to use verbatim memories to suppress the output of false memories, false memories tend to increase with age. Memory research has confirmed that children have more difficulty extracting gist from presented information and are poorer at generating links between different parts of an experience than adults (Bjorklund, 1987, 2005; Esposito, 1975).

Another theory that accounts for this developmental reversal is the Associative-activation Theory (AAT; Howe et al., 2009; Otgaar et al., 2013). AAT is a theory that explains the development of different types of false memories based in part on correlated developmental differences in knowledge base and automatic processing. The core point of AAT is that false memories arise due to spreading activation across meaning-connected information in memory. Processing of a word or concept results in an immediate and parallel spread of activation to related concept nodes in a person’s knowledge base (mental lexicon; Anderson, 1983; Collins & Loftus, 1975; Landauer & Dumais, 1997). During this spread of activation, concepts activate related concepts some of which were not experienced, leading to the production of false memories.

According to AAT, false memories increase with age because of changes in the structure, content, and speed of access to information in a child’s knowledge base. It is because of these changes that the strength and automaticity of associative activation increases with age, something which in turn catalyzes age-related increases in false memories from childhood through to adulthood (Howe, 2005; Howe & Wilkinson, 2011; Otgaar, Candel, Scoboria, & Merckelbach, 2010; Otgaar, Smeets, & Peters, 2012). False memories are more likely to develop as children mature because they gain knowledge and experience through formal and informal learning opportunities as well as through exposure to an increasingly complex array of experiences.

## Creating Developmental Reversals in Suggestion-Induced False Memories

In the current set of studies, we examined whether we could systematically alter (attenuate or reverse) developmental trends in suggestion-based false memories by using our understanding of how spontaneous memory illusions arise from meaning-connected memory information. Although spontaneous false memories are chiefly the result of endogenous processes (e.g., relying on meaning, spreading activation), suggestion-based false memories occur because of endogenous *and* exogenous (e.g., social influences) processes (also see Brainerd et al., 2008). The false memory paradigms that are used to evoke suggestion-based false memories do not contain the necessary ingredients that educe the age-related increases in false memories seen in the DRM paradigm. This is because these paradigms represent an amalgam of endogenous and exogenous (e.g., suggestive pressure) manipulations whereas what is needed is more of a focus on the endogenous (memory) processes of this task (e.g., meaning, spreading activation). In fact, such design changes should be relatively easy to implement. As Brainerd and colleagues (2011, p. 376) suggests, “the standard [*misinformation*] paradigm can readily be adjusted to fit the algorithm by instantiating the meaning of objects/events that are supplied to children as misinformation with many things that they actually experience during the initial phase of such an experiment, much like DRM lists.” So basically, during the initial phase of a misinformation experiment, participants should receive stimuli that are meaning connected. Following this, participants should be presented with misinformation that preserves the underlying meaning of the originally presented stimuli. The prediction is that this adapted misinformation procedure will likely result in an attenuated or even a reversed developmental trend like the one that is commonly found for spontaneous false memories.

Surprisingly, few researchers have followed these specific adjustments a priori to test whether false memories induced by suggestion using the misinformation paradigm can increase with age in a manner similar to spontaneous false memories (Ceci, Papierno, & Kulkofsky, 2007). Although certain studies (Connolly & Price, 2006; Fazio & Marsh, 2008; Principe, Guiliano, & Root, 2008; Ross et al., 2006) have found developmental reversal effects in other paradigms such as memory conformity, developmental reversals for implanted false memories such as those elicited by the misinformation are practically nonexistent. Considering that these false memories are perhaps one of the most relevant memory illusions in legal cases (Loftus, 2005), it is relevant to examine developmental reversals in the misinformation paradigm. More importantly, although some studies have found developmental reversal effects in other paradigms, they did not specifically check whether the presented stimuli contained related details and hence catalyzed false memory production; something that we will do in the present experiments (see below).

## The Current Experiments

In the current set of four experiments, we focused on the theoretical conditions necessary to alter developmental trends in children’s and adults’ suggestion-based false memories. Specifically, based on the tenets of both AAT and FTT, we modified the misinformation paradigm. Here, we presented participants with newly developed, forensically relevant video stimuli depicting events that contained meaning related details. We used videos because they are rich in perceptual detail and are frequently used in standard misinformation experiments (Loftus, 2005).

For Experiment 1, we constructed new videos that were based on principles found in the DRM paradigm (i.e., FTT’s gist extraction or AAT’s associative relatedness). These videos were then included in a misinformation paradigm and were presented to 6/7-year-olds, 11/12-year-olds, and adults. After the presentation of these videos, participants received misinformation about the videos and were then given a recognition task. In order to compare our findings with the DRM paradigm, participants were also given a standard DRM word list task. We conducted a second experiment in which our goal was to replicate the findings from Experiment 1 with a younger age group of 4/6-year-olds. For Experiments 3 and 4, we constructed yet another new video showing that the effects of Experiments 1 and 2 were not limited to the type of material used. In all of these experiments, the basic prediction was that when using meaning-connected stimuli, typical developmental trends in suggestion-induced false memories will be attenuated or even reversed.

Based on AAT and FTT, some specific predictions can be made about when standard developmental trajectories in suggestion-based false memories should become attenuated or reversed. For example, both AAT and FTT predict that just like the developmental reversals seen with DRM word lists, young children will be less likely to grasp the underlying meaning of these meaning-connected videos than older children and adults. Thus, when misinformation is provided about critical, nonpresented details, older children and adults will be more likely to accept this suggested misinformation than younger children. So, based on both AAT and FTT, our expectation was that when using meaning-connected videos, we should see a developmental reversal such that susceptibility to misinformation increases rather than decreases with age.

Interestingly, AAT also predicts additional circumstances under which developmental trends in suggestion-based false memories can become attenuated or reversed. In AAT, considerable weight is placed on the link between false memories and theme nodes. So, not only does spreading activation result in the activation of related concepts in one’s knowledge base, but it also leads to the activation of theme nodes: nodes that are related to the subset of concepts being activated (Arndt & Reder, 2003; Howe & Wilkinson, 2011; Otgaar et al., 2014). Theme nodes are part of associative memory networks and they too can give rise to false recollections. The idea is that material (e.g., DRM word lists) that activates fewer themes leads to more false memories than material that converges on multiple themes. The reason is that material containing fewer themes is more likely to activate that theme more quickly than when there are multiple themes. Moreover, the overall activation of this single theme may be greater than that for materials that have more themes because activation in this latter case is more dispersed across the many different themes. The consequence is that these higher levels of activation of a single theme catalyze false memories.

One way to examine this claim is to explore the effects of valence (i.e., neutral or negative) on developmental trends in false memory. Because the events that children encounter in forensic settings contain a number of important emotional elements, understanding how emotion modulates developmental patterns of (false) memories is important if findings are to be generalized to the legal field (also see Otgaar et al., 2013). If we look at the literature surrounding the effects of emotion on memory, then many studies have shown that emotional events boost memory performance (e.g., Mather, 2007; Phelps, 2006). This is especially true when these emotional events evoke arousal (and even stress), particularly during encoding and consolidation (Smeets, Otgaar, Candel, & Wolf, 2008). The main point here is that memory performance changes when emotional events evoke arousal. One idea is that arousal attracts attention to the evoking stimulus that in turn leads to binding of memory features, enhancing subsequent memory performance. It has also been shown that event valence (particularly negative valence) affects memory because emotional memories consist of well-integrated and dense networks of interrelated concepts (see, e.g., Talmi, 2013). In such a network, information is more likely to spread in a fast and automatic manner leading to better memory.

Because emotional events frequently boost memory accuracy, one would expect that this might also protect individuals from forming false memories for arousing and negative events. Although research concerning the relation between emotion and memory in adults suggests that negative events are less likely to foster false memories than neutral or positive events. (e.g., Corson & Verrier, 2007; Storbeck & Clore, 2005), recent studies have shown that negative stimuli can increase false memory rates over neutral stimuli (e.g., Howe, 2007; Howe, Candel, Otgaar, Malone, & Wimmer, 2010; Otgaar, Candel, & Merckelbach, 2008; Porter, Spencer, & Birt, 2003) and that developmental reversals are present for different emotionally charged materials (e.g., Howe et al., 2010; Quas et al., 2015). In AAT, this is expected because negative information converges on fewer theme nodes than neutral information. In FTT, this is predicted because it is easier to extract the gist of negative than neutral information (e.g., Brainerd, Holliday, Reyna, Yang, & Toglia, 2010). Thus, higher false memory rates are expected for negative than neutral materials. Although this is true for spontaneous false memories, how emotion interacts with developmental trends in suggestion-based false memories and whether developmental reversals in suggestion-based false memories are modulated by emotionally laden events has not been directly addressed. To investigate the influence of valence on false memory, children and adults in our experiments were presented with negative (as well as neutral stimuli).

## Experiment 1

For Experiment 1, we began by conducting a pilot study to develop new material. We presented participants with critical cue words (e.g., pistol, mailbox) and they had to provide response items related to these words. We used adult participants in this pilot work because research has shown that even if we had used material relevant specifically to children’s knowledge base, developmental patterns of false memory production are still quite robust (e.g., Metzger et al., 2008). The response items produced by adults were then used to construct two videos (i.e., a robbery and a postman bringing mail) in which the critical items were not included in the video.

For this pilot task, we developed a situational gist task where participants had to provide as many related response items as they could to each of a number of critical cue words in two different contexts, a cafeteria robbery and a postman delivering mail. These related response items were then used to develop two new videos. There were 20 participants (mean age = 21.50, *SD* = 2.89; 10 male) who each received a booklet in which 12 critical cue words were mentioned (e.g., pistol, money, glasses, mailbox). Importantly, for half of the critical cue words, it was stated that they had to come up with related response items when thinking about a robbery and for the other half of the items, they had to think about a scene in which a postman is bringing mail. For example, the exact instruction for the robbery scene was: “A cafeteria at a gym club is being robbed by an armed man. Try to come up with as many items (minimum of 5) that are related to this event.” They were given for the entire task (for all cue words) 15 min in order to generate as many related response items as they could.

The presentation order of the scenarios was counterbalanced across participants. For each of these scenarios, we selected three items to serve as central details for the video and three to serve as peripheral details. So, in total, there were six central and six peripheral items. Central items referred to details depicting the thematic content of the video (i.e., the robbery) whereas peripheral details were not specifically related to the theme of the video.

The participants produced many related response items (*N* = 449). For each of the critical items, we selected the five related items that were produced most often by participants. For example, for the central critical response word *pistol* in the robbery scene, participants came up with many different details (e.g., bullet), but most often mentioned were the following related items: *perpetrator, silver necklace, black jacket, hat,* and *black trousers.* These related items (but not the critical items were used as stimuli in the situational gist task) were included in the video of the robbery. These two new videos were presented to participants (6/7-year-olds, 11/12-year-olds, adults) in a modified misinformation paradigm. Our prediction was that when the misinformation was presented, older children and adults would be more likely to confuse the misinformation with the presented items in the videos than the younger children. Also, to compare our findings with the developmental trend in spontaneous false memories, we presented participants with both neutral and negative DRM word lists.

### Method

#### Participants

Eighty-five participants were included, with 23 6/7-year-olds (mean age = 6.70, *SD* = 0.56; 11 boys; range: 6–7), 30 11/12-year-olds (mean age = 11.47, *SD* = 0.63; 17 boys; range 11–12), and 32 adults (mean age = 21.38, *SD* = 3.53; 9 male; range 18–33). A power analysis with a power of 0.80 and a small to medium effect size (η_partial_^2^ = 0.06) resulted in a sample size of 80. For the child groups, we received school and parent permission to test 53 children (23 + 30). The remaining participants were tested in the adult pool. We tested five extra participants in case participants dropped out of the experiment, thus data collection was terminated after testing 85 participants. Children received a small gift and adults received a small financial reimbursement. All children had parental consent and assented on the day of testing. The study was approved by the standing ethical committee of the Faculty of Psychology and Neuroscience at Maastricht University.

#### Design

This experiment consisted of a 3 (Age: 6/7-year-olds vs. 11/12-year-olds vs. adults) × 2 (Misinformation: yes vs. no) × 2 (Detail: central vs. peripheral) × 2 (Valence: negative vs. neutral) mixed design with the latter three factors being within-participant variables.

#### Materials

##### Videos

Two videos were constructed based on the findings from the pilot experiment and that differed in emotional content. One video was emotionally negative and was about a robbery in a cafeteria in which a culprit enters the cafeteria and demands money from the people at the cash desk. The other video was neutral and showed a postman delivering mail and a man opening the door to receive the mail. In each video, 30 items were presented that were associatively related to six critical items (three central and three peripheral). Each video lasted for about 1 min and 10 s. During the videos, we ensured that all presented items were unambiguously visible.

##### Misinformation

Misinformation was presented in the form of an eyewitness account of the videos. This eyewitness account was audiotaped and played back to the participants. A research assistant that was not involved in this experiment acted as the eyewitness and was audiotaped when reading the account. The eyewitness accounts (control vs. critical) were the same for each participant. During the misinformation phase, the eyewitness mentioned six new critical items and 18 old items from the original video that were related to the critical items (for each critical item, three related items were presented from the original video). We also made a control version in which an eyewitness only mentioned the 18 original items without the new critical items.

##### Recognition task for the videos

Our recognition task was also audio taped and consisted of 48 correct items, 12 critical items, 12 nonpresented related items, and 48 nonpresented unrelated items. The recognition task was presented at a 5-s rate per item. Responses were recorded by the experimenter.

##### DRM lists

Five neutral (*bread, smoke, window, foot, sweet*) and five negative (*murder, punishment, cry, death, pain*) 10-word DRM lists were used in this experiment. The effectiveness of these lists in generating false memories has been demonstrated in previous studies (Howe et al., 2010; Otgaar & Candel, 2011; Otgaar et al., 2012) and list items were chosen from the Dutch word association norms (Van Loon-Vervoorn & Van Bekkum, 1991). List items were presented in order of associative strength to the nonpresented critical lure, from strongest to weakest. Using the Celex lexical database (Baayen, Piepenbrock, & Gulikers, 1995), it was ensured that the mean word frequency of neutral and emotional critical lures did not differ statistically, *t*(8) = 0.22, *ns*. Similarly, the mean backward associative strength between the neutral list items and their critical lures and the mean backward associative strength between the emotional list items and their critical lures did not vary statistically, *t*(8) = 1.69, *ns.*

##### Recognition task for the DRM lists

The recognition task for the DRM lists consisted of a total of 78 words where there were 40 correct items, 10 critical lures, 10 nonpresented related items, and 18 nonpresented unrelated items. The recognition task was audio taped and was presented at a 5-s rate per item.

#### Procedure

Children were tested individually in rooms at their elementary school and adults were tested at the university. Participants were informed that they were involved in a memory study and then received either the videos (misinformation paradigm) or the DRM lists first. Order of presentation of the stimuli was counterbalanced within each task. Our methodology was similar to the standard misinformation paradigm used in previous studies (Loftus, 2005). During presentation, participants saw the two videos on a computer screen and were told to pay close attention to them. After witnessing the videos, participants were given a nonverbal filler task (playing Tetris) for 3 min. Then, participants received audio taped misinformation about one of the videos and heard an audio taped control version with no misinformation about the other video. The order of the presentation of the misinformation and control tapes was also counterbalanced. Following exposure to the misinformation and the control tapes, participants had to perform a nonverbal filler task (playing Tetris) for 3 min. Next, they received a recognition task in which they were asked to recognize only those items that were presented in the videos. After the recognition task, all participants had to rate the emotionality of the videos on a 5-point Likert scale (1 = *very negative*, 5 = *very positive*). This was done to examine whether participants had noticed the valence of the videos. The negative video (*M* = 2.68, *SD* = 0.90) received statistically lower ratings than the neutral video (*M* = 3.55, *SD* = 0.75; *F*(1, 84) = 52.25, *p* < .001, η_p_^2^ = 0.38) indicating that the valence manipulation was effective. For the DRM word lists, participants received either the neutral lists first or the negative lists first. After the DRM word lists, participants were involved in a nonverbal filler task (playing Tetris). Then, participants were presented with the DRM recognition task.

### Results

As is customary in developmental research on spontaneous false memories, scores were corrected for possible response bias, a correction that leads to purer measures of hits and false memory (Brainerd & Reyna, 2007; Brainerd et al., 2008; Holliday, Brainerd, & Reyna, 2011). Specifically, scores of all experiments were transformed using the following signal detection parameters: *d*′ (memory discrimination) and *c* (response bias; Snodgrass & Corwin, 1988).^2^ Higher *d*′ values stand for high memory discrimination. Negative *c* values represent a liberal bias while positive *c* values refer to a conservative bias. Furthermore, when we analyzed age effects with three groups in all of our experiments, we used Bonferroni correction.

We begin by presenting the video data and then turn to the DRM lists. Finally, we present the analyses concerning the relationship between performance on the video task and performance on the DRM task. All data (raw and transformed) are reported in Tables 2, 3, 4, 5, 6, and 7.[Table-anchor tbl2][Table-anchor tbl3][Table-anchor tbl4][Table-anchor tbl5][Table-anchor tbl6][Table-anchor tbl7]

#### Video task

##### Hit rates

###### Memory discrimination

We performed a 3 (Age: 6/7-year-olds vs. 11/12-year-olds vs. adults) × 2 (Misinformation: yes vs. no) × 2 (Detail: central vs. peripheral) × 2 (Valence: negative vs. neutral) mixed ANOVA on the *d*′ hit rates. Our results yielded a statistically significant age effect, *F*(2, 82) = 3.83, *p* = .03, η_p_^2^ = 0.09. Post hoc tests revealed that adults had statistically higher *d*′ values than the older children (*p* = .03). All other comparisons were not statistically significant. We also found a statistically significant valence effect, *F*(1, 82) = 22.59, *p* < .001, η_p_^2^ = 0.22, with the negative video having statistically higher *d*′ values than the neutral video. Furthermore, central details had higher *d*′ values than peripheral details, *F*(1, 82) = 14.07, *p* < .001, η_p_^2^ = 0.15. All other effects were not significant, including those concerning the misinformation manipulation (see Table 3).

###### Bias

Like the discrimination index, we found a statistically significant Age effect, *F*(2, 78) = 7.10, p = .001, η_p_^2^ = 0.15, with post hoc comparisons showing that bias was most liberal in the 6/7-year-olds, but only statistically decreased for the 11/12-year-olds (*p* = .001). A statistically significant valence effect also emerged, *F*(1, 78) = 15.24, *p* < .001, η_p_^2^ = 0.16, with the neutral video having more liberal bias scores than the negative video. Furthermore, bias scores were more liberal for the peripheral than central details, *F*(1, 78) = 8.27, *p* = .005, η_p_^2^ = 0.10 (Table 4).

#### False memory

##### Memory discrimination

A similar series of statistical analyses was performed on the corrected false memory scores (critical items). First, the most important analysis pertained to whether susceptibility to misinformation increased with age. We indeed found evidence for this. Our analyses revealed a significant Valence × Age × Misinformation interaction, *F*(2, 79) = 8.26, *p* = .001, η_p_^2^ = .17. Additional tests showed the following results. For the negative video, there was a significant Age × Misinformation interaction, *F*(2, 79) = 3.83, *p* = .03, η_p_^2^ = .09, with simple effects showing that the susceptibility to misinformation (suggestion-based) false memories increased significantly with age; see Table 2). That is, for both types of details, we found that adults (central: *M* = 4.99, *SD* = 6.01; peripheral: *M* = 6.30, *SD* = 3.60) and older children (central: *M* = 2.35, *SD* = 5.72; peripheral: *M* = 6.39, *SD* = 2.96) had significantly higher *d*′ values rates than younger children (central: *M* = 0.48, *SD* = 2.66; peripheral: *M* = 5.43, *SD* = 5.39; *p* < .05). The difference between 11/12-year-olds and adults was not significant (*p* = 1.00; Table 3).

For the neutral video, we also found a significant Age × Misinformation interaction, *F*(2, 79) = 3.18, *p* = .047, η_p_^2^ = .08. Further analyses showed that like the negative video, the propensity to suggestion-based false memories increased from the 6/7-year-olds (*M* = 4.98) to the 11/12-year-olds (*M* = 5.55; *p* < .05), but this difference fell short of statistical significance. For the participants who did not receive misinformation, only 6/7-year-olds (*M* = 5.50) had statistically higher discrimination values than 11/12-year-olds (*M* = 2.42; *p* = .01).

Second, we found a significant Age × Detail × Valence interaction, *F*(2, 79) = 8.51, *p* < .001, η_p_^2^ = .18. For the neutral video, results yielded a significant Age × Detail interaction, *F*(2, 79) = 5.19, *p* = .008, η_p_^2^ = .12. Here, additional analyses showed that for the central details, 6/7-year-olds (*M* = 7.75, *SD* = 4.36) and adults (*M* = 4.71, *SD* = 4.95) differed significantly in their *d*′ values (*p* = .04). Older children (*M* = 5.40, *SD* = 3.79) did not differ statistically from the other groups (*p*s > .05). For the peripheral details, there were no statistically significant differences (*p* = 1.00).

For the negative video, we found a statistically significant Age × Detail interaction, *F*(2, 79) = 3.61, *p* = .03, η_p_^2^ = .08, with simple effects tests showing that *d*′ values for the peripheral details were higher than *d*′ values for the central aspects for younger (peripheral: *M* = 7.52, *SD* = 5.25; central: *M* = 2.16, *SD* = 3.22); *F*(1, 21) = 31.17, *p* < .001, η_p_^2^ = .60, and older children (peripheral: *M* = 5.95, *SD* = 4.23; central: *M* = 2.49; *SD* = 4.49); *F*(1, 28) = 13,05, *p* < .001, η_p_^2^ = .32, but there were no significant differences for adults, *F*(1, 30) = 1.74, *p* = .20, η_p_^2^ = .06.

##### Bias

Our analysis found a significant Valence × Age × Misinformation interaction, *F*(2, 79) = 6.76, *p* = .002, η_p_^2^ = 0.15. When we performed additional tests, we found the following. For the negative video, we found a significant Age × Misinformation, *F*(2, 79) = 6.24, *p* = .004, η_p_^2^ = 0.14. When misinformation was provided, 6/7-year-olds (*M* = −4.72) had statistically lower liberal bias scores than the 11/12-year-olds (*M* = −0.60, *p* = .02) while the reverse was true when no misinformation was given. Here, 6/7-year-olds (*M* = 0.48) were more conservative than adults (*M* = −3.12, *p* = .01).

For the neutral video, we found a main effect of misinformation, *F*(1, 79) = 5.69, *p* = .02, η_p_^2^ = 0.07, in which the presentation of misinformation led to more liberal bias values (*M* = −2.97) than when no misinformation was introduced (*M* = −1.24). All other effects were not statistically significant (see also Table 4).

#### False alarms for related and unrelated nonpresented items

##### Memory discrimination

We also executed analyses on the *d*′ values of the nonpresented related items. We found an Age × Valence interaction, *F*(2, 78) = 3.71, *p* = .03, η_p_^2^ = 0.09. Follow-up tests revealed that for the neutral video, adults (*M* = −1.64) did not differ in their discrimination values from 11/12-year-olds (*M* = −2.10) and 6/7-year-olds (*M* = −1.10) while the two child groups did differ (*p* = .03). For the negative video, discrimination values did differ between adults (*M* = −0.78) and 6/7-year-olds (*M* = 0.31; *p* = .03), but not for 11/12-year-olds (*M* = −1.04; *p* = 1.00).

##### Bias

For the bias scores, we only found an age effect, *F*(2, 78) = 8.08, *p* = .001, η_p_^2^ = 0.17, with adults (*M* = −6.50) differing statistically between 6/7-year-olds (*M* = −4.97) and 11/12-year-olds (*M* = −5.44; *p* = .004) while the latter two did not differ (*p* = 1.00).

When we focused on the raw scores of the unrelated items, we found that the older children had statistically higher unrelated scores than the adults, *F*(2, 82) = 4.01, *p* = .02, η_p_^2^ = .09.

#### DRM task

##### Hit rates

###### Memory discrimination

We also examined how participants performed on the DRM paradigm. A 3 (Age: 6/7-year-olds vs. 11/12-year-olds vs. adults) × 2 (Valence: negative vs. neutral) repeated measures ANOVA on the transformed DRM hits yielded a statistically significant age effect, *F*(2, 82) = 9.83, *p* < .001, η_p_^2^ = .19. Post hoc analyses showed that for both types of lists, adults (*M* = 3.49) had statistically higher discrimination values than 11/12- (*M* = 1.40) and 6/7-year-olds (*M* = 1.32; *p* = .001). The two child groups did not statistically differ (*p* = 1.00). All other effects were not statistically significant (see Table 6).

###### Bias

No statistical effects emerged (all *p*s > .05; Table 7).

#### False memory

##### Memory discrimination

An analysis of the discrimination values of the critical nonpresented items showed a significant Age × Valence interaction, *F*(2, 82) = 6.87, *p* = .002, η_p_^2^ = .14. Additional analyses revealed the following. First, for both types of lists, false memories increased with age, with adults and 11/12-year-olds having higher discrimination values than the youngest age group (*p*s < .05). Adults and 11/12-year-olds also differed in their level of false memory susceptibility (*p* = .04). Second, we found that negative false memories were more likely to be produced in 6/7- and 11/12-year-olds relative to neutral false memories (*p*s < .01). For adults, this difference was not significant (*p* = .34; Table 6)

##### Bias

A statistical Age × Valence interaction was found, *F*(2, 82) = 6.87, *p* = .002, η_p_^2^ = .14. Simple effects showed that bias scores for negative false memories were less liberal in 6/7- and 11/12-year-olds relative to bias scores for neutral false memories (*p*s < .01). For adults, this difference was not significant (*p* = .34; Table 7).

#### False alarms for related and unrelated nonpresented items

##### Memory discrimination

Analyses of the nonpresented related items revealed a significant valence effect, *F*(1, 82) = 31.21, *p* < .001, η_p_^2^ = .28, with higher discrimination values for the negative related items than for the neutral related items. All other effects were not statistically significant.

##### Bias

A significant age effect was observed, *F*(2, 82) = 7.48, *p* < .001, η_p_^2^ = .15, with post hoc tests showing that only adults (*M* = −5.48) had statistically more liberal bias scores than the 11/12-year-olds (*M* = −2.45; *p* = .001). We also found a valence effect, *F*(1, 82) = 25.61, *p* < .001, η_p_^2^ = .23, with more liberal bias scores for the neutral than the negative related items. The interaction was not significant.

When we analyzed the unrelated items, we found that the older children (*M* = 0.23, *SD* = 0.16) had statistically higher unrelated scores than the adults (*M* = 0.09, *SD* = 0.08); *F*(2, 82) = 7.07, *p* < .001, η_p_^2^ = .15.

#### Misinformation-DRM correlational analyses

Finally, we were interested in whether DRM false memories would be related to suggestion-based false memories. The correlations in all experiments were computed on discrimination scores. Interestingly, a partial correlational analysis in which we looked at four correlations (correlations between DRM and video false memories) and in which we controlled for age found that neutral DRM false memories were statistically related to neutral and negative suggestion-induced false memories, respectively (*r* = .24, *p* = .03; *r* = .38, *p* < .001). Likewise, we found that negative DRM false memories were statistically related to neutral and negative suggestion-induced false memories (*r* = .24, *p* = .03; *r* = .23, *p* = .04).

### Discussion

The results of this experiment showed that developmental reversals do exist in suggestion-based false memories. Specifically, when certain theoretically prescribed conditions pertain (i.e., when critical items in a misinformation paradigm are semantically related to the presented items during the study phase), suggestion-induced false memories increased with age. That is, we found that memory discrimination increased with age, a clear developmental reversal effect. This pattern was most evident for the negative suggestion-based false memories. Although suggestion-based false memories for neutral videos also increased with age, this increase fell short of significance. Thus, like the spontaneous DRM-based false memories in this experiment, suggestion-based false memories were more likely in older children and adults than in younger children.

In the current design, we ensured that during the study phase, participants encountered details that were associatively related to nonpresented critical items. Then, during the misinformation phase, misinformation was presented about these nonpresented critical items. When participants subsequently received a recognition task, older children and adults were more likely to associatively relate these critical items with the originally presented items in the videos than the younger children. This resulted in a developmental reversal of the usual trend in suggestibility research, one that showed that older children and adults were more likely to develop suggestion-based false memories than younger children.

For both the negative and neutral videos, however, we did not find that this reversal continued into adulthood. Specifically, in the negative video, we found no differences between 11/12-year-olds’ and adults’ false memory levels and for the neutral video we found that adults’ false memory levels were lower than the 11/12-year-olds’ false memory rates. For the negative video, our findings are in line with our DRM findings in that older children and adults did not differ in their false memory propensity. A possible reason for not finding a difference between 11/12-year-olds and adults is that children of this age activate related concepts in as rapid and automatic a manner as adults.

As mentioned, for the neutral video, we found that adults’ false memory rates were reduced compared to 11/12-year-olds’ false memory rates. There are other studies showing that adults’ false memory rates can be lower than children’s (Otgaar et al., 2012; Wimmer & Howe, 2010). Although speculative, researchers have noted that these age discrepancies in false memory formation might be tied to individual and population variations in false memory vulnerability (Bouwmeester & Verkoeijen, 2010). Despite these discrepancies, we still found that under circumstances that primarily rely on gist extraction (FTT) or associative activation (AAT), both spontaneous and suggestion-based false memories follow a positive monotonic trajectory between younger and older children.

Also, at a descriptive level, neutral false memory rates were slightly higher than negative false memories in young children. So, although all participants perceived the negative video as statistically more negative than the neutral one, this did not translate in statistically higher false memory rates for the negative video as what would be expected (Howe et al., 2010). One possibility is that associative networks for these videos differed between age group and that younger children had a more extensive knowledge base for the neutral than the negative video. Therefore, additional studies should examine whether participants’ knowledge base might vary between different types of emotional material.

However, although our DRM lists were specifically constructed to differ with regard to valence, our videos could have differed in terms of arousal. This is important because we know that arousal also drives changes in false memory rates (e.g., Brainerd et al., 2011). There are also other dimensions that might have differed between our videos (e.g., relatedness, familiarity) that could have potentially affected our results. It is therefore relevant to urge caution when interpreting our emotional false memory effects. To circumvent this issue, for our next second experiment, we opted to include only the negative video.

Interestingly, we found that both negative and neutral DRM false memories were positively related to the development of both negative and neutral suggestion-based false memories. This implies that similar processes, such as associative activation, are involved in developmental trends in both domains. Up until now, developmental studies linking DRM false memories with false memories for more complex realistic events have not been conducted (see Brainerd et al., 2008). Although there are studies that have found positive correlations between DRM false memories and false memories for autobiographical events in children and adults (e.g., Otgaar et al., 2012), these studies are somewhat limited. Our study presents a hitherto unreported finding suggesting that whatever the type of false memory (e.g., spontaneous or suggestion-induced) similar endogenous processes seem to play a role in developmental patterns of these false memories.

We also explored whether our effects might interact with the type of detail (peripheral vs. central). The reason behind this is that for emotional events particularly, central details are better remembered than peripheral details (Thijssen, Otgaar, Meijer, Smeets, & de Ruiter, 2012). However, in our experiment, developmental reversal effects were observed for both types of detail thereby demonstrating the robustness of this developmental reversal effect.

## Experiment 2

Because the results of Experiment 1 provide novel yet tentative evidence showing developmental reversals in suggestion-induced false memories with meaning-connected stimuli, we attempted to replicate these results in a new sample using a more straightforward procedure. To begin, because adults did not differ significantly from older children in terms of false memory propensity, we only included children in Experiment 2 and added an extra group of younger 4- to 6-year-olds. Because this latter group has often been found to be highly vulnerable to suggestive influences (Ceci & Bruck, 1993), it was important to see whether developmental reversals occurred at this younger age. Second, because our developmental effects seemed to be more pronounced for the negative than neutral video, we only used the negative video in this experiment. Third, because we obtained significant developmental reversals in the misinformation condition (as predicted), we eliminated the control condition in which no misinformation was presented.

## Method

### Participants

Seventy participants were involved in this experiment (4/6-year-olds: *n* = 18, mean age = 4.83, *SD* = 0.44, range 4–6; 7/9-year-olds: *n* = 21, mean age = 7.33, *SD* = 0.78, range 7–9; 10/12-year-olds: *n* = 31, mean age = 10.81, *SD* = 0.65, range 10–12). A power analysis with a power of 0.80 and a medium effect size (η_partial_^2^ = 0.13) resulted in a sample size of 69. Data collection was terminated after 70 children received consent to participate. We tested an extra participant in case children dropped out of the experiment. The goal was to test equal numbers of children in each age group, but the number of children was based on obtaining parental consent (and hence we had an unequal number of children in the groups). We stopped data collection when our total desired sample size was met. Children’s parents had provided consent for their child’s participation in the study and all children assented on the day of participation. All children received a small present for their participation. The study was approved by the standing ethical committee of the Faculty of Psychology and Neuroscience at Maastricht University.

### Design

The current experiment made use of a 3 (Age: 4/6-year-olds vs. 7/9-year-olds vs. 10/12-year-olds) × 2 (Detail: central vs. peripheral) split-plot design with the latter factor being within participant.

### Materials

We used the exact same negative video, misinformation (negative), DRM lists, and DRM recognition task as in Experiment 1. The only exception was the recognition task for the video.

Our recognition task was also audio taped and consisted of 24 correct items, six critical items, six nonpresented related items, and 24 nonpresented unrelated items. Items on the recognition task were presented at a 5-s rate. Responses were recorded by the experimenter.

### Procedure

Children were tested individually in rooms at their primary school. They received either the video (misinformation paradigm) or the DRM word lists (DRM paradigm) first. We counterbalanced the order of the presentation of the stimuli within each paradigm. A similar procedure was used as in Experiment 1 except that all participants received misinformation about the video.

### Results

As in Experiment 1, all scores on the video and DRM procedure were transformed using memory discrimination (*d*′) and response bias (*c*). All data ([un]corrected video and DRM Tasks) are reported in Tables 8, 9, 10, 11, 12, and 13 (video and DRM tasks).[Table-anchor tbl8][Table-anchor tbl9][Table-anchor tbl10][Table-anchor tbl11][Table-anchor tbl12][Table-anchor tbl13]

#### Video task

##### Hit rates

###### Memory discrimination

When we performed a 3 (Age: 4/6-year-olds vs. 7/9-year-olds vs. 10/12-year-olds) × 2 (Detail: central vs. peripheral) repeated measures ANOVA, we found no significant interaction, *F*(2, 66) = 1.14, *p* = .87, η_p_^2^ = .004, no significant effect for detail, *F*(1, 66) = 0.01, *p* = .92, η_p_^2^ = .00, but there was a significant age effect, *F*(2, 66) = 6.34, *p* = .003, η_p_^2^ = .16. Post hoc tests revealed that the only 10/12-year-olds (*M* = 7.73) had statistically higher memory discrimination rates relative to the youngest child group (*M* = 5.29; *p* = .004; Table 9)

###### Bias

No statistical effects emerged when performed analyses on the response bias scores (all *p*s > .05; Table 10).

#### False memory

##### Memory discrimination

An analysis of the false memory rates did not reveal a significant interaction, *F*(2, 66) = 1.42, *p* = .25, η_p_^2^ = .04, but we did find a statistically significant age effect, *F*(2, 66) = 7.61, *p* = .001, η_p_^2^ = .19. As in Experiment 1, we found a developmental reversal effect. That is, 10/12-year-olds (*M* = 6.94) and 7/9-year-olds (*M* = 6.16) had statistically higher memory discrimination values than the 4/6-year-olds (*M* = 3.50; *p*s < .05). Results also demonstrated a significant main effect of detail, *F*(1, 66) = 14.26, *p* < .001, η_p_^2^ = .18, with discrimination values being higher for peripheral false items than for central false items (see Table 9).

##### Bias

When we performed an analysis on the response bias scores, we only found a statistically reliable detail effect, *F*(1, 66) = 12.73, *p* = .001, η_p_^2^ = .16, with bias values being less liberal for peripheral than central false items. All other effects were not statistically significant (see Table 10).

#### False alarms for related and unrelated nonpresented items

When we conducted our analyses for nonpresented (un)related items, no statistically significant effects emerged.

#### DRM task

##### Hit rates

###### Memory discrimination

A repeated measures ANOVA on the discrimination values of the hit rates revealed a statistically significant effect of valence, *F*(1, 66) = 9.76, *p* = .003, η_p_^2^ = .13. Specifically, discrimination values were higher for the negative than neutral presented items. All other effects were not statistically significant (see Table 12).

###### Bias

Again, we only found a statistically significant valence effect, *F*(1, 66) = 8.75, *p* = .004, η_p_^2^ = .12, with less liberal bias scores for the negative than neutral presented items (see Table 13).

#### False memory

##### Memory discrimination

We also performed an analysis on the discrimination values of the false memory rates. We found no significant interaction, *F*(2, 66) = 1.19, *p* = .31, η_p_^2^ = .04, but we did find a developmental reversal effect, *F*(2, 66) = 8.33, *p* = .001, η_p_^2^ = .20. Post hoc analyses showed that the 10/12-year-olds (*M* = 3.93) had significantly higher false memory rates than the 7/9-year-olds (*M* = 1.87; *p* = .02) and the 4/6-year-olds (*M* = 1.07; *p* = .001). The 7/9-year-olds and 4/6-year-olds did not differ statistically in terms of false memory propensity (*p* = 1.00). We also found that negative false memories had higher bias scores than neutral false memories, *F*(1, 66) = 11.64, *p* = .001, η_p_^2^ = .15; Table 12.

##### Bias

When we focused our analysis on the bias scores, we found that 10/12-year-olds (*M* = 3.14) had statistically more conservative bias scores than the 7/9- (*M* = .62; *p* = .002) and 4/6-year-olds (*M* = .08; *p* < .001); *F*(2, 66) = 11.03, *p* < .001, η_p_^2^ = .25. Furthermore, we found that bias scores were more conservative for the negative than neutral critical items, *F*(1, 66) = 32.79, *p* < .001, η_p_^2^ = .33; Table 13.

#### False alarms for related and unrelated nonpresented items

##### Memory discrimination

An analysis of the *d*′ values of the nonpresented related items revealed no significant interaction, *F*(2, 66) = 1.32, *p* = .27, η_p_^2^ = .04. We did find that negative related items had higher discrimination scores than neutral related items, *F*(1, 66) = 5.80, *p* = .02, η_p_^2^ = .08. No age effect was detected, *F*(2, 66) = 0.12, *p* = .89, η_p_^2^ = .004.

##### Bias

We only found that negative related items had lower liberal bias scores than neutral related items, *F*(1, 66) = 6.02, *p* = .02, η_p_^2^ = .08.

No statistical effects emerged for the unrelated items, *F*(2, 66) = .65, *p* = .52, η_p_^2^ = .02.

#### Misinformation-DRM correlational analyses

We were also interested in whether suggestion-based false memories were associated with the DRM illusion. When performing four partial correlational analyses controlling for age, we found that negative, *r* = .32, *p* = .001, DRM false memories were statistically related to the central suggestion-based false memories.

### Discussion

The primary goal of Experiment 2 was to replicate the findings from Experiment 1 with younger children. That is, we wanted to show that suggestion-based false memories increased with age when there was an emphasis on associatively related meaning-based processing. This experiment showed convincingly that this developmental reversal did occur with very young children when using a video-misinformation experiment. Indeed, again, we found that memory discrimination scores increased with age and the same pattern was evident for the untransformed scores. Together with Experiment 1, our results demonstrate that when suggestion-based false memories rely on spreading activation among associatively related concepts (and themes) that developmental reversals occur in a manner similar to that found for children’s spontaneous false memories.

Of course, one might argue that our findings are limited inasmuch as we used the same materials in both of our experiments. In order to demonstrate that our findings are not confined to these materials and that they can be replicated using other video-material, we conducted two additional experiments. Because our findings suggest that negative stimuli resulted in stronger developmental reversals than neutral material, we decided to only use a new negative video, one that is also relevant in forensic settings. Again, we had to pilot this new material using a situational gist task. We also explored whether the presentation of different types of misinformation affected the developmental reversal effect. That is, in Experiments 3 and 4, we not only presented misinformation in the form of eyewitness testimony as in our previous experiments, but for half of the participants we also presented the same misinformation during a suggestive interview. We did this because previous studies have found that children are more susceptible to misinformation than adults if it is delivered by authority figures (e.g., adults), particularly in an interview setting (see Ceci & Bruck, 1993).

Thus, in these final two experiments, participants could receive one of two versions of misinformation. As before, half of the participants were presented with misinformation in the form of another person’s eyewitness testimony. Recall that this minimizes the impact of direct social influences on false memory formation (i.e., there is no authority asking suggestive questions) and hence, we anticipated developmental reversals in this group. In the other group, the exact same misinformation was presented but in the form of an interview with the participants. Here, because participants were confronted with misinformation in a social context, one might not expect developmental reversals. This is because such a context includes suggestive pressure by an authority, something that might lead to the standard developmental trends in suggestion-induced false memories (i.e., an age-related decrease in false memories).

## Experiment 3

For this experiment, a pilot study was conducted to develop new material. Participants received critical cue words (e.g., pistol, money) and they had to come up with related response items to these words. Like Experiment 1, we used adult undergraduates from the psychology faculty of Maastricht University in this pilot study because, as mentioned, previous research has shown that even if materials are normed specifically for children, developmental patterns of false memory formation are still quite robust (e.g., Metzger et al., 2008). The response items produced by adults were then used to construct a video of a bank robbery in which the critical cue words were not included in the video.

For this pilot task, we again used a situational gist task where participants had to provide as many related response items as they could to each of a number of critical cue words in a single context, namely, a bank robbery. These related response items were then used for the construction of the video. There were 38 participants (mean age = 28, *SD* = 3.56; 20 male) who each received a booklet in which 10 critical cue words were mentioned (e.g., pistol, money, laptop). For the entire task, they were given 15 min to produce as many related items as they could. The exact instruction for the bank robbery scene was: “A bank is being robbed by an armed man. Try to come up with as many items (minimum of 5) that are related to this event.”

The participants collectively produced many related response items (*N* = 1,773). Of the 10 critical cue words, five were selected for which participants produced the most related response items (i.e., pistol, money, laptop, brochures, numberticket-dispensor). For each of the five critical cue words we selected the five related items that were produced most often by participants. For example, for the critical cue word *pistol*, participants most often mentioned the following related response items: *loud noise, criminal, bullets, black clothing,* and *balaclava.* These related response items were included in the eventual video of the bank robbery and the critical cue words were left out of the video. Central critical cue words that were used as misinformation were *pistol* and *money* and peripheral cue words were *laptop*, *brochures*, *numberticket-dispensor*. This video was then used as part of a misinformation paradigm in which we examined whether this new material would change the usual developmental trend in suggestion-induced false memories and whether it would lead to the same developmental reversals in suggestion-based false memories as our first two experiments.

### Method

#### Participants

In this study, 44 7/8-year-olds (mean age = 7.16, *SD* = 0.37; range 7–8; 23 boys) and 42 adult participants (mean age = 20.79, *SD* = 2.60; range 18–28; six men) were involved. A power analysis with a power of 0.80 and a medium effect size (η_partial_^2^ = 0.13) resulted in a sample size of 84 (Faul et al., 2007). As 44 children received parental consent to participate, we stopped with data collection after testing a total of 86 participants. These age groups were selected because they differ significantly in terms of false memory propensity (Brainerd et al., 2008). Children were recruited from elementary schools and could only participate if they received parental consent and assented on the day of testing. Children received a small present for their involvement. Adults were undergraduates from the Faculty of Psychology and Neuroscience and the Faculty of Health, Medicine, and Life Sciences. They received a monetary award (7.50 euro) or course credits for their participation. The study was approved by the standing ethical committee of the Faculty of Psychology and Neuroscience at Maastricht University.

#### Materials

##### Misinformation

Misinformation was presented in two forms: as an eyewitness account or in an interview. The eyewitness account was audiotaped and played back to the participants. The eyewitness mentioned the five critical items and 20 old items from the original video that were related to the critical items (for each critical item, four related items were presented from the original video). In the other version, participants were subjected to a short interview in which they were asked 25 questions. Five questions contained misinformation including the not-presented critical items (e.g., “What was the color of the pistol of the bank robber?”). The other 20 questions pertained to old items that were presented during the video (e.g., “Was the color of the balaclava the same as his jacket?”). Participants were asked to respond to each question and had to guess if they did not know the answer.

##### Recognition task for the video

The 50-item recognition task consisted of five critical items, 20 presented items (four presented for each critical item), five related but not-presented items (one for each critical item), and 20 unrelated but not-presented items. Items on the recognition task were presented at a 5-s rate. Responses were recorded by the experimenter.

##### DRM lists

Participants heard five negative DRM word lists each containing 10 words. These were the same negative DRM lists as in Experiments 1 and 2. The words were related to critical lures that were the nonpresented items (e.g., *death, punishment*). These lists have proven to effectively elicit spontaneous false memories (Howe et al., 2010; Otgaar et al., 2010).

##### Recognition task for the DRM lists

The 45-item recognition task consisted of five critical lures, 20 presented items (four from each list), five related but not-presented items (one for each critical lure), and 15 unrelated but not-presented items. The recognition task was presented at a 5-s rate for each item. Responses were recorded by the experimenter.

#### Design and procedure

We used a 2 (Age: 7/8-year-olds vs. adults) × 2 (Condition: eyewitness account vs. interview) × 2 (Detail: central vs. peripheral) mixed design with the latter factor being within participant. Participants were randomly assigned to the conditions (eyewitness account: 21 children and 21 adults; interview: 23 children and 21 adults).

Children were tested in quiet rooms at their schools and adults’ test sessions took place in lab rooms at the university. Participants were told that they would witness a video and that certain questions were going to be asked about the video. Participants then had to look at the video. After this, a 2-min filler task (underline the letters R and P on a piece of text) was presented to participants. Next, participants received misinformation in the form of an eyewitness account or a suggestive interview. One day later, participants received the recognition test for the video. This final stage happened after a day in which participants’ memory was tested for the event because misinformation effects are stronger after a delay (Higham, 1998). Following this, participants studied five DRM lists, were given a 2-min filler task, and were then given the DRM recognition task.

### Results

#### Video task

##### Hit rates

###### Memory discrimination

Data were transformed as in the previous experiments. All data are reported in Tables 14, 15, 16, 17, 18, and 19 (video and DRM tasks). A 2 (Age: 7/8-year-olds vs. adults) × 2 (Condition: eyewitness account vs. interview) × 2 (Detail: central vs. peripheral) ANOVA was conducted on the corrected hit rates. A statistically significant three-way interaction was obtained, *F*(1, 83) = 12.77, *p* = .001, η_partial_^2^ = .13. Simple effects analyses revealed that for the interview, a statistically significant age effect was observed, *F*(1, 83) = 13.63, *p* = .001, η_partial_^2^ = .25, for the central items with higher discrimination values for the adults than children. This effect was absent in the eyewitness account condition, *F*(1, 83) = 1.50, *p* = .23, η_partial_^2^ = .04. For the peripheral items, there was a statistically significant age effect, *F*(1, 83) = 20.57, *p* < .001, η_partial_^2^ = .20, with adults having elevated *d*′ values relative to children (see Table 15).[Table-anchor tbl14][Table-anchor tbl15][Table-anchor tbl16][Table-anchor tbl17][Table-anchor tbl18][Table-anchor tbl19]

###### Bias

For the bias scores, we again found a statistically reliable three-way interaction, *F*(1, 83) = 13.01, *p* = .001, η_partial_^2^ = .14. Follow-up tests showed the following. For the central items, bias scores were more conservative in children than in adults, but only in the eyewitness condition, *F*(1, 42) = 4.64, *p* = .04, η_partial_^2^ = .10. For the interview condition, this was not statistically significant. For the peripheral items, there was a general statistical age effect, *F*(1, 83) = 4.24, *p* = .04, η_partial_^2^ = .05, with children having lower liberal bias scores than adults (see Table 16).

#### False memory

##### Memory discrimination

When we performed similar analyses on the *d*′ values of the false recognition rates (critical items), the following results emerged. Here, we found a statistically significant Age × Detail interaction, *F*(1, 83) = 6.44, *p* = .01, η_partial_^2^ = .07. As expected, our results showed that the standard developmental trend in suggestion-induced false memories was significantly altered such that it resulted in a developmental reversal effect when misinformation was introduced. Specifically, simple effect analyses found the following. For peripheral items, we found that children were less vulnerable to misinformation than adults, *F*(1, 85) = 5.63, *p* = .02; η_partial_^2^ = .06, a finding that illustrates a developmental reversal effect. For the central items, the results showed no developmental differences between children’s and adults’ false memory propensity, *F*(1, 83) = 1.89, *p* = .17, illustrating an attenuation of the usual developmental pattern in suggestion-induced false memories (see Table 15).

##### Bias

Our analysis only revealed a statistically significant Age × Detail interaction significant, *F*(1, 83) = 5.25, *p* = .03, η_partial_^2^ = .06. Additional tests found that only for the peripheral critical items, bias scores were statistically more conservative in children than in adults, *F*(1, 83) = 4.24, *p* = .04; η_partial_^2^ = .05. This difference was not statistically significant for the central critical items, *F*(1, 85) = 0.61, *p* = .44; η_partial_^2^ = .01l (see Table 16).

#### False alarms for related and unrelated nonpresented items

##### Memory discrimination

We also looked at *d*′ values of related and unrelated items. We found a statistically significant age effect, *F*(1, 83) = 5.87, *p* = .02, η_partial_^2^ = .07, with adults (*M* = 2.78) having higher discrimination scores than children (*M* = 1.18). Our analysis also found that discrimination scores were higher for the central than peripheral related items, *F*(1, 83) = 18.22, *p* < .001, η_partial_^2^ = .18.

##### Bias

Our analysis found statistically lower liberal bias scores for children (*M* = −4.45) than adults (*M* = −6.26); *F*(1, 83) = 6.20, *p* = .02; η_partial_^2^ = .07. Furthermore, lower liberal bias scores were found for the central than peripheral related items, *F*(1, 83) = 17.02, *p* < .001; η_partial_^2^ = .17.

For the unrelated items, we found that children had statistically higher false alarm rates than adults, *F*(1, 83) = 10.54, *p* = .002, η_partial_^2^ = .11.

#### DRM task

##### Hit rates

###### Memory discrimination

We conducted an ANOVA on the discrimination scores for the DRM lists. This analysis showed a statistically significant age effect, *F*(1, 85) = 16.05, *p* < .001, η_partial_^2^ = .16, where adults (*M* = 4.42) had higher scores than children (*M* = 2.28); *F*(1, 85) = 5.63, *p* = .02; η_partial_^2^ = .06 (see Table 18).

###### Bias

No statistical effects emerged (see Table 19).

#### False memory

##### Memory discrimination

Analyses of false memories showed the standard developmental reversal effect, *F*(1, 85) = 25.38, *p* < .001, η_partial_^2^ = .23, with adults having higher discrimination scores than children (see Table 18).

##### Bias

We found that adults had more conservative bias scores than children, *F*(1, 85) = 7.39, *p* = .008; η_partial_^2^ = .08 (Table 19).

#### False alarms for related and unrelated nonpresented items

##### Memory discrimination

For related items, we also found that adults (*M* = .43) had higher acceptance rates than children (*M* = −1.36); *F*(1, 85) = 5.75, *p* = .02, η_partial_^2^ = .06.

##### Bias

No statistical effects emerged.

For unrelated items, false alarm rates were statistically higher for children than adults, *F*(1, 85) = 21.50, *p* < .001, η_partial_^2^ = .20.

#### Misinformation-DRM correlational analyses

When we examined whether video false memories were related to DRM false memories, three partial correlational analyses (controlling for age) showed that video false memories for the peripheral items were statistically related to DRM false memories, *r* = .29, *p* = .006.

### Discussion

Once again, our findings from Experiment 3 provide clear evidence that using our meaning-modified misinformation paradigm results in reversals of the usual developmental trends in suggestion-induced false memories. That is, when children and adults were confronted with stimuli containing meaningful, associatively related details, and were subsequently presented with associatively related misinformation (preserving the meaning of the event) about critical, nonpresented items, children were less prone to accepting that misinformation than adults, at least for peripheral information. Although we had no strong predictions concerning developmental trends for false memories for central and peripheral items, we did find an attenuation (central items) and a reversal (peripheral items) of suggestion-induced false memory development. Specifically, our analysis showed that as expected, when exposed to misinformation, children were not the most vulnerable to false memories and were sometimes the least vulnerable.

Because developmental reversals in suggestion-induced false memories represents a relatively new field of scientific inquiry, we conducted Experiment 4 in an attempt to further replicate our previous findings (see, e.g., Simons, 2014) as well as see whether developmental reversals can be produced for both central and peripheral items. In order to examine this, we tested a larger age span and now specifically focused on development trends in false memories in children. That is, we tested 4/5-year-olds, 7/8-year-olds, and 11/12-year-olds. We did not test adult participants in this study because false memory formation is often not different between 11/12-year-olds and adults (Otgaar et al., 2014).

## Experiment 4

### Participants

In this experiment, we included 52 4/5-year-olds (mean age = 4.60, *SD* = 0.50; range 4–5; 26 boys), 55 7/8-year-olds (mean age = 7.62, *SD* = 0.50; range: 7–8; 29 boys), and 51 11/12-year-olds (mean age = 11.51, *SD* = 0.51; range: 11–12; 24 boys). A power analysis resulted in a sample size of 68 participants. All children had parental consent and received a small present for their involvement. We received parental and school consent to test 158 children and hence, tested all children. Although equal numbers of children in each group were desired, consent for child participation meant in that the numbers of children differed somewhat in each age group. The study was approved by the standing ethical committee of the Faculty of Psychology and Neuroscience at Maastricht University.

### Materials

The exact same materials were used here as in Experiment 3.

### Design and Procedure

We used a 3 (Age: 4/5-year-olds, 7/8-year-olds, 11/12-year-olds) × 2 (Condition: eyewitness account vs. interview) × 2 (Detail: central vs. peripheral) mixed design with the latter factor constituting a within-participant variable. Participants were randomly assigned to the different conditions (eyewitness account: 27 4/5-year-olds, 29 7/8-year-olds, 26 11/12-year-olds; interview: 25 4/5-year-olds, 26 7/8-year-olds, 25 11/12-year-olds). A similar procedure was implemented as in Experiment 3.

### Results

#### Video task

##### Hit rates

###### Memory discrimination

Data were transformed as in the previous experiments (see Tables 20, 21, 22, 23, 24, and 25 for the video and DRM data). We conducted a 3 (Age: 4/5-year-olds, 7/8-year-olds, 11/12-year-olds) × 2 (Condition: eyewitness account vs. interview) × 2 (Detail: central vs. peripheral) ANOVA on hit rates. A statistically significant age effect was detected, *F*(2, 144) = 23.37, *p* < .001, η_partial_^2^ = .25. Post hoc comparisons showed that 4/5-year-olds (*M* = 2.72) had statistically lower discrimination scores than the 7/8- (*M* = 5.97) and 11/12-year-olds (*M* = 2.72; all *p*s < .001). We also found a significant detail effect with central items having higher values than peripheral ones, *F*(1, 144) = 46.09, *p* < .001, η_partial_^2^ = .24. All other effects were not statistically significant (see Table 21).[Table-anchor tbl20][Table-anchor tbl21][Table-anchor tbl22][Table-anchor tbl23][Table-anchor tbl24][Table-anchor tbl25]

###### Bias

We only found a statistically significant detail effect with the most conservative bias scores for the central items, *F*(1, 144) = 45.00, *p* < .001, η_partial_^2^ = .24 (see Table 22).

#### False memory

##### Memory discrimination

No significant three-way interaction emerged, *F*(2, 148) = 1.06, *p* = .35, η_partial_^2^ = .01. However, as predicted, we found a developmental reversal effect. That is, a statistically significant Age × Detail interaction emerged, *F*(2, 148) = 6.45, *p* = .002; η_partial_^2^ = .08, where simple effects analyses revealed that for both central and peripheral items, false memories increased with age—central: *F*(2, 149) = 10.33, *p* < .001; η_partial_^2^ = .12; peripheral: *F*(2, 148) = 21.19, *p* < .001; η_partial_^2^ = .22. However, for central items, 4/5-year-olds and 11/12-year-olds did not differ in terms of false memory rates (*p* = .052) although this effect was significant for 4/5-year-olds and 7/8-year-olds (*p* < .001). For the peripheral items, false memories increased with age with 4/5-year-olds having lower false memory rates than 7/8-year-olds and 11/12-year-olds (*p*s <.001). All other effects were not statistically significant (see Table 21).

##### Bias

For bias scores, the analysis also demonstrated a significant Age × Detail interaction, *F*(2, 148) = 6.33, *p* = .002, η_partial_^2^ = .08. Follow-up tests revealed that for both central and peripheral critical items, an age effect was observed—central: *F*(2, 149) = 3.39, *p* = .04, η_partial_^2^ = .04; peripheral: *F*(2, 148) = 4.62, *p* = .01, η_partial_^2^ = .06. However, for central critical items, post hoc comparisons showed that only the 4/5-year-olds and 7/8-year-olds differed somewhat in their bias scores; albeit not significant (*p* = .054). For peripheral critical items, 11/12-year-olds had more conservative bias scores than the youngest age group (*p* = .01). All other effects were not significant (see Table 22).

#### False alarms for related and unrelated nonpresented items

##### Memory discrimination

For *d*′ values of the related items, we only found that 4/5-year-olds (*M* = 1.04) had lower discrimination values than the 11/12-year-olds (*M* = 1.56; *F*(2, 145) = 3.47, *p* = .03; η_partial_^2^ = .05). We also found that central related items were better recognized than peripheral related items, *F*(1, 145) = 21.15, *p* < .001; η_partial_^2^ = .13. All other effects were not significant.

##### Bias

We found that only the youngest children (*M* = −2.46) had statistically lower liberal bias scores than the 11/12-year-olds (*M* = −4.90); *F*(2, 145) = 5.60, *p* = .005, η_partial_^2^ = .07. Our analysis also showed that bias scores were less liberal for central than peripheral related items, *F*(1, 145) = 20.98, *p* < .001, η_partial_^2^ = .13. All other effects were not significant.

For the unrelated items, 4/5-year-olds had statistically higher false alarm rates than 7/8-year-old sand 11/12-year-olds, *F*(2, 143) = 20.79, *p* < .001; η_partial_^2^ = .23.

#### DRM task

##### Hit rates

###### Memory discrimination

A univariate ANOVA was performed on the *d*′ values of the DRM hit rates. A statistically significant age effect emerged, *F*(2, 150) = 19.50, *p* < .001; η_partial_^2^ = .21. Post hoc tests showed that although 11/12-year-olds (*M* = 3.20) and 7/8-year-olds (*M* = 2.26) remembered more correct items than 4/5-year-olds (*M* = .63; *p*s < .001), the difference between 7/8-year-olds and 11/12-year-olds was not significant (*p* = .07; Table 24).

###### Bias

A significant age effect was observed, *F(*2, 150) = 9.62, *p* < .001, η_partial_^2^ = .11, showing that 4/5-year-olds (*M* = 1.48) had more conservative bias scores than the 7/8- (*M* = −.08) and 11/12-year-olds (*M* = −.34; *p*s < .002; Table 25).

#### False memory

##### Memory discrimination

For *d*′ values of false memories we found a statistically significant main effect of age, *F*(2, 151) = 124.11, *p* < .001; η_partial_^2^ = .45. Post hoc tests revealed a developmental reversal effect in that 11/12-year-olds (*M* = 4.56) had higher false memory rates than 7/8-year-olds (*M* = 2.73) who in turn had higher false memory levels than 4/5-year-olds (*M* = .81; all *p*s < .05; Table 24).

##### Bias

No age effect was found (see Table 25).

#### False alarms for related and unrelated nonpresented items

##### Memory discrimination

We found that 11/12-year-olds (*M* = .85) had higher discrimination values than 7/8-year-olds (*M* = −.80); *F*(2, 151) = 4.27, *p* = .01, η_partial_^2^ = .05.

##### Bias

The youngest age group (*M* = .67) had more conservative bias scores than the 7/8-year-olds (*M* = −3.14) and 11/12-year-olds (*M* = −2.64); *F*(2, 151) = 13.39, *p* < .001, η_partial_^2^ = .15.

For the unrelated items, we found that the youngest group had statistically higher false alarm rates than the 7/8-year-olds and 11/12-year-olds, *F*(2, 151) = 43.59, *p* < .001; η_partial_^2^ = .37.

#### Misinformation-DRM correlational analyses

Three partial correlational analyses (controlling for age) revealed that video false memories for central information were statistically related to the formation of DRM false memories, *r* = .19, *p* = .02.

### Discussion

The findings of Experiment 4 again convincingly demonstrate that false memories in a suggestibility paradigm can increase with age under circumstances that focus on the meaning of an event. In this experiment, we even found stronger evidence for a developmental reversal effect inasmuch as reversals were obtained for both central and peripheral details. Of course, this occurred only when we included 4/5-year-olds. Moreover, in this study, we found that for both the interview and eyewitness account, susceptibility to misinformation increased with age.

In both Experiments 3 and 4, we showed that when using new material, standard age-related trajectories in suggestion-induced false memories were attenuated or even reversed. Although we found in Experiment 3 that this effect was most pronounced for the peripheral items, in Experiment 4, we found developmental reversal effects for both central and peripheral items, irrespective of misinformation condition (interview vs. eyewitness statements). Although we expected that an interview would introduce elements of social pressure, we did not find that this altered developmental trends in suggestion-induced false memories.

### Cross-Experiment Analysis of Developmental Reversals

For each experiment, we identified how many developmental reversal effects for the video and DRM tasks were predicted and checked how many we found. For Experiment 1 (video task), we only predicted developmental reversals when misinformation was provided (*n* = 4; neutral-central, neutral-peripheral, negative-central, negative-peripheral). We found reversal effects in all four of them. For the DRM task, we predicted two reversal effects (neutral and negative) and we also found reversals for both of them. For Experiment 2 (video task), we expected to find two reversal effects (central and peripheral) and reversal effects were found for both. For the DRM task, we expected two reversal effects (neutral and negative) and two were also detected. For Experiment 3 (video task), four developmental reversals were predicted (interview-central, interview-peripheral, eyewitness account-central, eyewitness account-peripheral), and we found evidence for two reversal effects (interview-central, interview-peripheral). For the other two, we found attenuation effects. For the DRM task, we expected one developmental reversal effect and we found evidence for this prediction. For Experiment 4 (video task), we expected four reversal effects (see Experiment 3) and we found evidence for all of them. For the DRM task, one reversal effect was predicted and this was also found. Taken together, for the video and DRM tasks, we find developmental reversal effects in 90% of the cases (18/20). If we only focus on the suggestion-induced false memories, then we find evidence for reversal effects in 86% (12/14) of the cases.

## General Discussion

Our experiments were designed to answer a simple question: Does the typical developmental trend in suggestion-induced false memories (i.e., age-related decline in false memory) change when (associatively or semantically) related information is used as misinformation? The answer is yes. Four experiments showed that under theoretically prescribed conditions (where misinformation is associatively related to the originally studied information) that older children and adults were more vulnerable to false memories than younger children. That is, in contrast to the usual suggestibility effects in childhood, younger children were not more vulnerable to false memory production than older children and adults. Indeed, we even found evidence for a developmental reversal effect in suggestion-based false memories. That we found similar results in different age groups (4- to 12-year-olds and adults) reinforces the argument that under conditions in which people have to rely on thematic, associative activation, younger children are not more susceptible to false memories than older children and adults and are even sometimes the least vulnerable to false memory formation. Moreover, across the four experiments we used a number of newly created materials and found evidence for changes in the typical developmental trend in suggestion-induced false memories across these materials.

If we look more closely at the analyses of age effects in false memory across experiments (DRM and misinformation), we find that 90% (*n* = 18) of these analyses showed clear evidence for developmental reversals. If we only focus on the misinformation experiments, 86% (*n* = 12) show developmental reversal effects (see cross-experiment analysis of developmental reversals). This clearly shows that our experiments were quite successful in demonstrating that when using meaning-connected material, developmental trends in children’s susceptibility to misinformation and false memories can be altered and can even be reversed so that they increase with age. If we compare this with the overview of studies on developmental false memory effects (see Figure 1a), it is obvious that our experiments reveal the malleability of suggestion-based false memory development.

Importantly, we found that our developmental effects in suggestion-based false memories were found mainly in terms of false recognition of critical items. Similar developmental trends were less pronounced in other variables (e.g., related items). This is in line with previous research on developmental trends in spontaneous false memories (Brainerd et al., 2008) and reinforces our argument that our procedure specifically resulted in spreading activation to critical items and did not spill over onto less related items. Furthermore, our developmental reversal effects seemed to go hand in hand with younger children having lower hit rates than older children and adults, another standard finding in memory development research (Brainerd et al., 2008).

In Experiments 3 and 4, we also explored whether the presentation of different types of misinformation (social pressure in an interview format vs. eyewitness testimony) affected developmental trends in false memory differently. The reasoning behind this was that external influences such as social pressure might not load on any endogenous processes (associative activation, gist extraction) and hence, lead to the standard age-related decrease in suggestion-based false memories. In Experiments 3 and 4, we found that both social pressure and eyewitness testimony led to developmental reversal effects. Of course, this issue warrants further examination, but it might imply that even social pressure is not a reliable predictor for causing younger children to assent more to misinformation than older children and adults.

### Links to Theories of Memory Development

Our studies were derived in large measure from the tenets of AAT (Howe et al., 2009) and it turns out that our findings are consistent with this theory. Recall that in AAT, false memories arise out of automatic associative activation in one’s knowledge base and that false memories increase with age because as one’s knowledge base expands and is restructured (also see Ceci et al., 2007), spreading activation becomes more automatic (Howe et al., 2009). The presentation of associatively related information during the misinformation phase increased children’s associative activation resulting in either no differences between children’s and adults’ false memory rates or a developmental increase in false memories. Thus, when misinformation included the related nonpresented details, it was the older children (and adults) who were most likely to associate these details within their knowledge base and form false memories.

Of course, it is important to acknowledge that other false memory theories are also able to explain the current findings. For example, in FTT, developmental reversals in suggestion-induced false memories would be predicted when gist-related information was provided during the misinformation phase. Because younger children were less likely to get the gist from the videos than older children (and adults), and when misinformation was provided that included the related nonpresented items, older children (and adults) were more likely to associate this with the presented details and developed more false memories than younger children.

Another aim of the present research was to explore whether valence would interact with developmental trends in suggestion-induced false memories. That is, the affect-as-information hypothesis (Corson & Verrier, 2007) predicts that negative experiences do not lead as easily to false memories as positive ones because people attend more to item-specific details in negative events, something that lowers false memory production. However, both AAT and FTT assume that it is easier to extract the underlying meaning of negative events than more mundane (or positive) events, because negative events evoke networks of more strongly interrelated nodes. Information activation is therefore more likely to spread throughout networks of negative than neutral material, increasing false memory rates. Indeed, we found that negative materials led to more false memories than neutral material and that younger children were less likely to produce false memories than older children and adults for both the neutral and negative material. Thus, in line with the work on valence and DRM lists (Howe et al., 2010), our experiments also revealed that valence does not interact with developmental trends in suggestion-induced false memories. However, one should interpret our emotional false memory effects with caution as it is unclear whether our video material (in Experiment 1) differed in valence, arousal, or on even more dimensions (e.g., familiarity). Future studies could attempt to replicate the present findings with stimuli controlling for factors such as valence and arousal.

Finally, our results are related to a developmental-representational theory that specifies that differences in mental representations of experiences drive memory development and that these differences explain reversals in memory development. One important discovery in this area concerns age improvements in metamemory (i.e., introspection of the contents of memory). This research has shown that metamemory abilities protect people from the acceptance of false information (Ghetti, 2008). Indeed, one might argue that falsely recognizing the critical lure during the recognition task could be due to poor source monitoring in that participants mistakenly recognize the critical lure as being part of the original event while it was presented as part of the misinformation. It is true that misinformation effects are often explained in terms of source misattributions (e.g., Loftus, 2005). However, poor source monitoring is unable to explain the developmental reversal effects found in the current experiments. That is, research shows that younger children have poorer source monitoring abilities than older children and adults (Lindsay, Johnson, & Kwon, 1991). Based on this, one would expect that younger children would be more vulnerable to misinformation than older children and adults. Because we found the opposite pattern in our experiments, it is likely that our results are better explained in terms of developmental changes in spreading activation, changes in knowledge base, or the ability to extract gist.

### Links to Other Studies of False Memory

One may wonder whether our findings are novel. Developmental reversals have surfaced in several other memory paradigms besides the DRM paradigm (Candel, Memon, & Al-Harazi, 2007: memory conformity; Connolly & Price, 2006: suggestibility; Ceci et al., 2007: misinformation; Goswick, Mullet, & Marsh, 2013: stories; Lyons, Ghetti, & Cornoldi, 2010: causal narratives; Odegard, Cooper, Lampinen, Reyna, & Brainerd, 2009: group play; Otgaar & Smeets, 2010: survival processing; Principe et al., 2008: rumor-mongering; Ross et al., 2006: eyewitness identification; Sloutsky & Fisher, 2004: categorized lists; see for an overview, Brainerd, 2013). For example, Candel, Memon, and Al-Harazi (2007) showed that in free recall, memory conformity effects were stronger in 11/12-year-olds than in 6/7-year-olds. Also, Principe, Guiliano, and Root (2008) found developmental reversal effects in a paradigm measuring rumor-mongering. Furthermore, Ross et al. (2006) found that adults made more identifications of an innocent bystander than children. However, there are several critical dimensions present in the current project that differentiates it from this previous research, making the present results novel.

First, as seen earlier, research into developmental reversals in the *misinformation paradigm* is extremely limited and developmental reversals have not been extensively examined for implanted false memories (i.e., misinformation-based false memories). This is surprising as this paradigm has been at the heart of studies linking false memories with the legal arena. Indeed, the misinformation paradigm is frequently regarded as one of the most important paradigms for studying false memories (Loftus, 2005). More importantly, none of the studies listed above followed the recommendations put forward by Brainerd et al. (2011) who suggested that researchers should study developmental reversals in the misinformation paradigm. That is, Brainerd et al. (2011) argued that developmental reversals in the misinformation paradigm can be revealed when children and adults receive material containing associatively related details that are then presented with misinformation that preserves the underlying meaning of the event. We are the first using such a procedure and in line with theories as FTT and AAT, we find developmental reversal effects for suggestion-induced false memories. Second, and as has been articulated by Ceci Papierno, and Kulkofsky (2007) and Brainerd et al. (2011), the present findings are novel because they have been predicted from an a priori position and are closely based on theoretical mechanisms found in both FTT and AAT; something that has not been done before to the extent we have examined it in this article (see below).

It is true that our findings are in line with Ceci et al.’s (2007) study in that these researchers also used an adapted misinformation paradigm. However, our experiments add new perspectives to this work. First, we extend Ceci et al.’s (2007) findings as we have used different and more forensically relevant (videos) materials, ones that also differed in emotion. Second, we conducted four misinformation experiments using our adapted protocol and showed developmental reversals across all of them. Indeed, these reversals in suggestion-induced false memories are quite robust, something that has not been demonstrated prior to the research reported in this article. Third, reversals in suggestion-induced false memories have not been examined from an a priori perspective. Indeed, Ceci, Fitneva, and Williams (2010, p. 465) acknowledge that work in this area is important because “[s]uch reversals, albeit rare, present a serious challenge to theory, and past accounts of their occurrence have been post hoc and have not led to a priori predictions of when younger and older children’s performance will be similar or reversed.” Finally, in contrast to previous studies, a novel element of our experiments is that we compared false memories obtained with our new material with DRM false memories. In this way, we could check whether mechanisms underlying DRM false memories (i.e., associative activation) also played a role in the elicitation of suggestion-induced false memories. As expected, we found evidence that susceptibility to suggestion-induced false memory was positively linked to DRM false memory illusions, independent of age.

The crucial message from the experiments presented here is that the assumption that children’s testimonial accuracy is necessarily inferior to that of adults’ is untenable. Indeed, simply by changing the nature of the materials, we found that older children (and adults) produced more false memories than younger children. Thus, we have demonstrated that the validity of such an assumption depends on a number of considerations, ones that derive from theoretical principles concerning the role of meaning-connected information in events and how this information interacts with memory development generally and the formation of false memories specifically.

The focus of our article was on an examination of developmental trajectories of suggestion-induced false memories that were grounded in semantic activation. Of course, this limits the generalizability of our findings to other false memories that are the result of external influences such as social pressure or forced confabulation. Also, one could argue that our findings are silent about developmental trends in false memories for autobiographical, real-life experiences. Although this limitation needs to be taken seriously, there is evidence showing developmental reversal effects in other eyewitness paradigms such as rumor-mongering and group conformity (e.g., Candel et al., 2007; Principe et al., 2008). However, it is still important to be cautious about how far we can generalize our findings and acknowledge the continuing debate as to whether memory illusions based on semantic activation are related to other types of (false) memories (e.g., Gallo, 2010; Ost et al., 2013; Otgaar, Verschuere, Meijer, & Van Oorsouw, 2012).

### Links to Other Domains

Our results have implications for domains other than memory development. For example, our experiments are in line with the accumulating body of research in cognitive development that is also showing that younger children sometimes outperform older children and adults. Indeed, younger children are better at distinguishing foreign sounds than older children and adults (Kuhl, 2004; Werker, Yeung, & Yoshida, 2012). Also, younger children are superior in coming up with alternative ways to use tools than older children (Defeyter & German, 2003). Finally, Gopnik, Griffiths, and Lucas (2015) showed that unusual abstract causal principles were better learned by younger than older children.

A likely candidate for a common mechanism for these counterintuitive developmental patterns is age-related changes in one’s knowledge base. Indeed, according to Gopnik et al. (2015), acquiring new knowledge might result in being less flexible for new ideas. Furthermore, they reasoned that although a dense and well-integrated knowledge base might consist of many interrelated connections, these connections do not leave room for exploratory behavior. For the field of memory, our experiments show that such a dense and well-integrated knowledge base can also be disadvantageous because it gives rise to false recollections.

The implication here is that more focus should be placed on understanding the role of knowledge base in developmental studies. There are several specific areas that might benefit from such a focus. For example, in educational contexts, considerable weight is placed on learning new material and integrating it with one’s current knowledge base. In order for this to occur, it would be useful to know the current status of students’ knowledge in order to tailor the new material so that it can be easily integrated when learning takes place (see also Roediger, 2013). Like Gopnik et al. (2015), it would be relevant to assess whether for certain concepts and tasks, older children experience more learning difficulties than younger children.

Another area that might advance from a focus on one’s knowledge base is the forensic context. The lesson from the current experiments is that a child’s age can no longer be used as a predictor of their reliability as an eyewitness. Indeed, in many criminal cases, expert witnesses regularly (falsely) assume that young children are more apt to produce most kinds of memory errors, whether they arise spontaneously or due to suggestion-induced pressures (for a recent case, see Brackmann, Otgaar, Sauerland, & Jelicic, 2015). However, there are perhaps other forensic ramifications of our findings. For example, although not done at present, one interesting possibility might be to examine whether the DRM paradigm (referring to one’s knowledge base) is a valid and reliable method of indexing a person’s susceptibility to form spontaneous false memories in an interrogation setting. Eyewitnesses, victims, and suspects are occasionally tested on their vulnerability to suggestive pressure (e.g., Gudjonsson, Sigurdsson, Bragason, Newton, & Einarsson, 2008), but there is virtually no empirical knowledge about whether the DRM paradigm could be a useful tool in an interrogation setting as well (Brainerd et al., 2011).

## Conclusion

To recap, we have shown that when using well-specified theoretical principles, developmental trends in false memories can be manipulated. Although it is frequently the case that misinformation effects are more pronounced in younger than older children and adults, we found that developmental trends in these suggestion-based memory illusions can be reversed. Indeed, across all of the misinformation experiments reported in this article, susceptibility to suggestion did not always decrease with age, and that under certain specific conditions, older children (and adults) were more prone to suggestion-induced false memories than younger children.

## Figures and Tables

**Table 1 tbl1:** Developmental Studies on the Misinformation Effect

				Result		
Study	Age (years)	Event	Form of misinformation	Younger more suggestible than older	Younger less suggestible than older	No difference
Cohen & Harnick (1980)	9, 12, college students	Video about petty crime	Misleading questions	x		
King & Yuille (1987)	6, 9, 11, 16	Staged event	Misleading questions	x		
Ceci, Ross, & Toglia (1987), Exp. 1	3–12	Auditory story + slides about a girl’s day at school (no crime)	Misleading information	x		
Ornstein, Gordon, & Larus (1992)	3, 6	Physical examination	Misleading questions	x		
Oates & Shrimpton (1991)	4–12	Blood collection, interaction with stranger	Misleading questions	x		
Marin, Holmes, Guth, & Kovac (1979)	5–22	Staged event (interaction between experimenters, no crime)	Misleading questions			x (only one leading question)
Duncan, Whitney, & Kunen (1982), Exp. 2	7, 9, 11, college students	Slides with short *Star Wars* episodes	Misleading verbal information		x	
Flin, Boon, Knox, & Bull (1992)	6, 10, adults	Staged event (talk about foot hygiene, no crime)	Misleading questions			x
Rudy & Goodman (1991)	4, 7	Interaction with stranger, watching interaction (playing board game)	Misleading questions	x actions that occurred		x overall
Saywitz, Goodman, Nicholas, & Moan (1991)	5, 7	Physical examination (genital and nongenital)	Misleading questions	x		
Perner & Wimmer (1988)	2–4	Narrative about mother interacting with children (no crime)	Embedded in narrative (only one item)	x		
Ackil & Zaragoza (1995)	7, 9, 11, college students	Video about camp experiences (no crime)	Embedded in narrative	x		
Welch-Ross, Diecidue, & Miller (1997)	3–5	Narrative about day of a girl	Misleading questions	x (4 min delay)		x (1 week delay)
Hünefeldt, Rossi-Arnaud, & Furia (2009)	4–7	Cartoon-video	Misleading questions	x		
Hünefeldt, Lucidi, Furia, & Rossi-Arnaud (2008)	4–7	Cartoon-video	Misleading questions	x		
Kulkovsky & Klemfuss (2008)	2–5	Staged event baking cookies	Misleading questions	x		
Bright-Paul, Jarrold, & Wright (2008)	3–7	Slide show about theft	Embedded in narrative	x		
Quas et al. (2007)	3, 5	Playing alone in laboratory	Biased interviewer/misleading questions	x (misleading questions)	x (only in free recall single interview, long delay)	
Roebers, Howie, & Beuscher (2007)	6–8	Video about treasure hunt	Misleading questions	x		
Melinder, Endestad, & Magnussen (2006)	3, 6	Video showing children playing together	Misleading questions	x		
Roebers & Schneider (2005)	6, 7, 8, adults	Video about treasure hunt	Misleading questions	x		
Roebers, Gelhaar, & Schneider (2004)	5–10	Staged event, video or slide show about visit of magician	Misleading questions	x		
Alexander et al. (2002)	3–7	Vaccination	Misleading questions	x		
Gobbo, Mega, & Pipe (2002), Exp. 1	3, 5	Participation, observation or narration about playing with salt-dough	Misleading questions	x (immediate interview)		x (interview 1 week later)
Roebers & Schneider (2002)	6, 8, 10	Video about money theft and treasure hunt	Misleading questions	x		
Roebers, Bjorklund, Schneider, & Cassel (2002)	5, 7, 10, adults	Video about theft of a bike	Misleading questions	x		
Newcombe & Dour (2001)	5, 6	Story accompanied by pictures about pet	Embedded in narrative	x		
Templeton & Wilcox (2000)	3, 4, 6, adults	Video showing *Sesame Street*	Embedded in narrative	x (original test)		x (modified test)
Otgaar, Candel, Smeets, & Merckelbach (2010)	4–5, 8–11	Instructed interaction with a puppet	Erroneous feedback	x (commission error)		x (omission error)

**Table 2 tbl2:** Means and Standard Deviations (in Parentheses) of Raw Hits, and False Alarms to Critical Lures, and Nonpresented Related Items as a Function of Age, Type of Detail, and Valence (Experiment 1)

	6/7-year-olds				11/12-year-olds				Adults			
	Misinformation		Control		Misinformation		Control		Misinformation		Control	
	Central	Periph	Central	Periph	Central	Periph	Central	Periph	Central	Periph	Central	Periph
Hits												
Neutral	.63 (.16)	.46 (.20)	.60 (.22)	.41 (.18)	.77 (.11)	.73 (.12)	.77 (.12)	.74 (.14)	.72 (.15)	.73 (.13)	.71 (.18)	.70 (.17)
Negative	.63 (.27)	.56 (.22)	.67 (.26)	.64 (.14)	.86 (.11)	.72 (.17)	.84 (.15)	.78 (.14)	.79 (.13)	.73 (.13)	.78 (.14)	.73 (.17)
CL												
Neutral	.70 (.33)	.30 (.30)	.79 (.34)	.27 (.25)	.80 (.21)	.58 (.24)	.65 (.15)	.29 (.33)	.46 (.38)	.29 (.11)	.49 (.24)	.25 (.15)
Negative	.12 (.17)	.58 (.34)	.42 (.25)	.83 (.22)	.46 (.32)	.74 (.24)	.37 (.20)	.69 (.28)	.48 (.39)	.56 (.27)	.33 (.27)	.46 (.34)
Related												
Neutral	.00 (.00)	.17 (.22)	.00 (.00)	.06 (.13)	.00 (.00)	.09 (.15)	.00 (.00)	.09 (.20)	.00 (.00)	.00 (.00)	.00 (.00)	.00 (.00)
Negative	.03 (.10)	.18 (.23)	.19 (.26)	.05 (.13)	.08 (.14)	.13 (.22)	.04 (.11)	.04 (.11)	.00 (.00)	.00 (.00)	.00 (.00)	.00 (.00)
Unrel	.02 (.03)		.02 (.02)		.05 (.06)		.03 (.04)		.01 (.03)		.02 (.02)	
*Note*. CL = critical lures; Periph = peripheral; Unrel = unrelated.												

**Table 3 tbl3:** Means and Standard Deviations (in Parentheses) of d′ Values of Hits, and False Alarms to Critical Lures, and Nonpresented Related Items as a Function of Age, Type of Detail, and Valence (Experiment 1)

	6/7-year-olds				11/12-year-olds				Adults			
	Misinformation		Control		Misinformation		Control		Misinformation		Control	
	Central	Periph	Central	Periph	Central	Periph	Central	Periph	Central	Periph	Central	Periph
Hits												
Neutral	3.91 (1.41)	3.24 (1.56)	3.94 (1.41)	3.39 (1.49)	3.53 (1.39)	2.97 (2.09)	3.55 (1.35)	3.02 (2.15)	4.18 (1.74)	4.13 (1.35)	4.75 (1.56)	4.57 (1.54)
Negative	3.79 (1.68)	3.83 (1.28)	3.81 (1.78)	3.91 (1.36)	3.88 (1.48)	3.26 (1.33)	3.93 (1.63)	3.65 (1.48)	4.91 (1.45)	4.65 (1.36)	4.31 (1.50)	4.16 (1.56)
CL												
Neutral	7.00 (4.80)	2.95 (4.13)	8.57 (3.88)	2.44 (2.80)	6.62 (4.41)	4.48 (3.85)	4.19 (2.69)	.65 (3.42)	3.81 (6.05)	3.50 (4.17)	5.60 (3.51)	3.61 (3.87)
Negative	.48 (2.66)	5.43 (5.39)	3.69 (2.99)	9.44 (4.50)	2.35 (5.72)	6.39 (2.96)	2.59 (3.47)	5.62 (5.07)	4.99 (6.01)	6.30 (3.60)	3.27 (3.38)	4.77 (5.35)
Related												
Neutral	−2.46 (1.51)	.65 (2.31)	−2.33 (1.52)	−.30 (1.26)	−3.29 (1.52)	−.89 (1.87)	−3.29 (1.55)	−1.12 (1.65)	−2.52 (1.50)	−1.27 (1.50)	−2.01 (1.38)	−.76 (1.38)
Negative	−.25 (1.55)	.95 (2.07)	1.01 (1.68)	−.50 (2.21)	−.78 (1.89)	−.75 (2.03)	−1.02 (2.40)	−1.45 (1.81)	−.28 (1.38)	−.76 (1.38)	−.79 (1.50)	−1.27 (1.50)
*Note*. CL = critical lures; Periph = peripheral.												

**Table 4 tbl4:** Means and Standard Deviations (in Parentheses) of C Scores of Hits, and False Alarms to Critical Lures, and Nonpresented Related Items as a Function of Age, Type of Detail, and Valence (Experiment 1)

	6/7-year-olds				11/12-year-olds				Adults			
	Misinformation		Control		Misinformation		Control		Misinformation		Control	
	Central	Periph	Central	Periph	Central	Periph	Central	Periph	Central	Periph	Central	Periph
Hits												
Neutral	−1.40 (.1.02)	−2.07 (.1.56)	−1.46 (1.30)	−2.02 (1.21)	−.54 (.1.06)	−1.10 (1.43)	−.52 (.1.15)	−1.05 (.146)	−.96 (.1.01)	−1.09 (1.00)	−1.04 (.1.19)	−1.41 (.78)
Negative	−1.76 (1.94)	−1.72 (1.14)	−1.50 (1.88)	−1.39 (1.01)	−.03 (1.18)	−.65 (1.14)	−.26 (.95)	−.54 (.97)	−1.08 (.91)	−1.33 (.90)	−.91 (.94)	−1.06 (.94)
CL												
Neutral	1.46 (4.32)	−2.59 (6.22)	.89 (5.31)	−5.24 (4.41)	1.41 (3.17)	−.73 (2.82)	−1.02 (2.32)	−4.56 (5.44)	−3.33 (4.75)	−3.65 (2.06)	−2.95 (2.72)	−4.56 (3.14)
Negative	−7.20 (4.20)	−2.25 (4.82)	−1.85 (4.79)	2.80 (3.68)	−2.61 (.3.58)	1.42 (4.71)	−2.81 (2.66)	.22 (4.26)	−3.56 (5.15)	−2.25 (3.76)	−3.87 (4.63	−2.38 (4.86)
Related												
Neutral	−4.65 (2.67)	−4.65 (2.67)	−5.60 (2.85)	−5.60 (2.85)	−4.96 (2.41)	−4.96 (2.41)	−5.19 (2.44)	−5.19 (2.44)	−6.49 (.75)	−6.49 (.75)	−6.75 (.69)	−6.75 (.69)
Negative	−4.85 (3.61)	−4.34 (3.15)	−4.29 (2.71)	5.80 (1.87)	−4.69 (2.06)	−4.66 (2.63)	−5.21 (1.11)	−5.64 (1.78)	−6.26 (.69)	−6.75 (.69)	−6.00 (.75)	−6.50 (.75)
*Note*. CL = critical lures; Periph = peripheral.												

**Table 5 tbl5:** Means and Standard Deviations (in Parentheses) of Raw DRM Hits, and False Alarms to Critical Lures, and Nonpresented Related Items as a Function of Age and Valence (Experiment 1)

	6/7-year-olds		11/12-year-olds		Adults	
	Neutral	Negative	Neutral	Negative	Neutral	Negative
Hits	.35 (.21)	.52 (.24)	.58 (.18)	.66 (.15)	.75 (.11)	.75 (.17)
CL	.30 (.29)	.51 (.31)	.61 (.23)	.79 (.21)	.69 (.27)	.67 (.25)
Related	.15 (.26)	.23 (.26)	.19 (.18)	.35 (.20)	.06 (.12)	.18 (.15)
Unrel	.18 (.20)		.23 (.16)		.09 (.08)	
*Note.* DRM = Deese/Roediger-McDermott; CL = critical lures; Unrel = unrelated.						

**Table 6 tbl6:** Means and Standard Deviations (in Parentheses) of d′ Values for DRM Hits, and False Alarms to Critical Lures, and Nonpresented Related Items as a Function of Age and Valence (Experiment 1)

	6/7-year-olds		11/12-year-olds		Adults	
	Neutral	Negative	Neutral	Negative	Neutral	Negative
Hits	1.06 (1.64)	1.55 (1.66)	1.38 (1.61)	1.62 (1.62)	3.54 (3.15)	3.44 (2.59)
CL	−.04 (2.67)	1.77 (2.92)	1.66 (1.86)	3.84 (3.29)	4.80 (3.56)	4.20 (3.85)
Related	−2.69 (4.11)	−1.24 (2.72)	−1.78 (3.32)	.37 (2.28)	−2.97 (3.13)	.02 (3.84)
*Note.* DRM = Deese/Roediger-McDermott; CL = critical lures.						

**Table 7 tbl7:** Means and Standard Deviations (in Parentheses) of C Values for DRM hits, and False Alarms to Critical Lures, and Nonpresented Related Items as a Function of Age and Valence (Experiment 1)

	6/7-year-olds		11/12-year-olds		Adults	
	Neutral	Negative	Neutral	Negative	Neutral	Negative
Hits	−1.40 (2.45)	−.89 (2.53)	−.37 (1.09)	−.13 (1.03)	−.47 (1.47)	−.57 (.147)
CL	−1.97 (4.74)	−.16 (4.54)	−.08 (1.78)	2.10 (3.41)	.79 (3.70)	.19 (3.67)
Related	−4.62 (5.06)	−3.71 (4.24)	−3.52 (3.43)	−1.38 (2.03)	−6.98 (3.29)	−3.98 (3.35)
*Note.* DRM = Deese/Roediger-McDermott; CL = critical lures.						

**Table 8 tbl8:** Means and Standard Deviations (in Parentheses) of Raw Hits, and False Alarms to Critical Lures, and Nonpresented Related Items as a Function of Age and Valence (Experiment 2)

	4/6-year-olds		7/9-year-olds		10/12-year-olds	
	Central	Peripheral	Central	Peripheral	Central	Peripheral
Hits	.56 (.23)	.62 (.22)	.74 (.17)	.71 (.18)	.81 (.12)	.82 (.13)
CL	.30 (.32)	.57 (.38)	.62 (.29)	.72 (.27)	.57 (.31)	.74 (.29)
Related	.11 (.23)	.13 (.26)	.17 (.17)	.17 (.28)	.13 (.19)	.19 (.24)
Unrel	.03 (.05)		.05 (.12)		.01 (.02)	
*Note.* CL = critical lures; Unrel = unrelated.						

**Table 9 tbl9:** Means and Standard Deviations (in Parentheses) of d′ Values for Hits, and False Alarms to Critical Lures, and Nonpresented Related Items as a Function of Age and Valence (Experiment 2)

	4/6-year-olds		7/9-year-olds		10/12-year-olds	
	Central	Peripheral	Central	Peripheral	Central	Peripheral
Hits	5.35 (2.55)	5.23 (2.61)	6.02 (2.84)	6.20 (2.65)	7.80 (2.85)	7.65 (2.61)
CL	1.89 (4.06)	5.10 (3.63)	5.62 (3.00)	6.71 (3.21)	6.19 (3.99)	7.68 (3.72)
Related	−.61 (.3.11)	.08 (4.63)	1.29 (3.03)	.72 (3.26)	1.52 (4.00)	2.61 (3.95)
*Note.* CL = critical lures.						

**Table 10 tbl10:** Means and Standard Deviations (in Parentheses) of C Values for Hits, and False Alarms to Critical Lures, and Nonpresented Related Items as a Function of Age and Valence (Experiment 2)

	4/6-year-olds		7/9-year-olds		10/12-year-olds	
	Central	Peripheral	Central	Peripheral	Central	Peripheral
Hits	−1.51 (2.96)	−1.68 (2.18)	−1.52 (2.32)	−1.34 (2.97)	−1.47 (2.41)	−1.62 (2.21)
CL	−3.73 (5.16)	−.85 (5.87)	−1.27 (3.55)	.34 (4.06)	−2.24 (3.08)	−.24 (4.37)
Related	−6.73 (5.74)	−6.05 (6.04)	−6.25 (4.31)	−6.82 (5.12)	−7.76 (3.28)	−6.67 (4.18)
*Note.* CL = critical lures.						

**Table 11 tbl11:** Means and Standard Deviations (in Parentheses) of Raw DRM Hits, and False Alarms to Critical Lures, and Nonpresented Related Items as a Function of Age and Valence (Experiment 2)

	4/6-year-olds		7/9-year-olds		10/12-year-olds	
	Neutral	Negative	Neutral	Negative	Neutral	Negative
Hits	.50 (.26)	.56 (.25)	.53 (.24)	.56 (.16)	.56 (.20)	.68 (.16)
CL	.41 (.32)	.52 (.31)	.40 (.29)	.64 (.24)	.63 (.30)	.86 (.17)
Related	.23 (.28)	.26 (.30)	.16 (.18)	.23 (.23)	.15 (.18)	.29 (.22)
Unrel	.28 (.26)		.23 (.22)		.21 (.14)	
*Note.* DRM = Deese/Roediger-McDermott; CL = critical lures; Unrel = unrelated.						

**Table 12 tbl12:** Means and Standard Deviations (in Parentheses) of d′ Values for DRM Hits, and False Alarms to Critical Lures, and Nonpresented Related Items as a Function of Age and Valence (Experiment 2)

	4/6-year-olds		7/9-year-olds		10/12-year-olds	
	Neutral	Negative	Neutral	Negative	Neutral	Negative
Hits	1.32 (1.89)	1.54 (1.90)	1.75 (2.26)	1.86 (2.35)	1.23 (1.48)	1.58 (1.31)
CL	.76 (2.52)	1.39 (3.09)	.84 (3.25)	2.91 (2.92)	2.70 (3.64)	5.17 (3.52)
Related	−1.63 (3.31)	−1.50 (3.44)	−2.09 (3.63)	−.77 (3.61)	−2.81 (3.34)	−.80 (3.22)
*Note.* DRM = Deese/Roediger-McDermott; CL = critical lures.						

**Table 13 tbl13:** Means and Standard Deviations (in Parentheses) of C Values for DRM Hits, and False Alarms to Critical Lures, and Nonpresented Related Items as a Function of Age and Valence (Experiment 2)

	4/6-year-olds		7/9-year-olds		10/12-year-olds	
	Neutral	Negative	Neutral	Negative	Neutral	Negative
Hits	−.68 (1.70)	−.50 (.166)	−.77 (1.62)	−.66 (1.39)	−.35 (.72)	−.00 (.76)
CL	−1.23 (4.72)	1.39 (3.09)	−1.68 (3.75)	2.91 (2.92)	1.11 (3.60)	5.17 (3.52)
Related	−3.56 (4.89)	−3.36 (4.17)	−4.60 (3.84)	−3.28 (3.52)	−4.40 (3.48)	−2.39 (2.88)
*Note.* DRM = Deese/Roediger-McDermott; CL = critical lures.						

**Table 14 tbl14:** Means and Standard Deviations (in Parentheses) of Raw Hits, and False Alarms to Critical Lures, and Nonpresented (Un)Related Items as a Function of Age (Experiment 3)

	7/8-year-olds		Adults	
	Central	Peripheral	Central	Peripheral
Hits	.86 (.13)	.63 (.21)	.89 (.11)	.76 (.11)
CL	.80 (.34)	.70 (.29)	.57 (.38)	.75 (.27)
Related	.42 (.32)	.16 (.23)	.35 (.34)	.16 (.24)
Unrel	.04 (.05)		.01 (.03)	
*Note.* CL = critical lures; Unrel = unrelated.				

**Table 15 tbl15:** Means and Standard Deviations (in Parentheses) of d′ Values for Hits, and False Alarms to Critical Lures, and Nonpresented Related Items as a Function of Age (Experiment 3)

	7/8-year-olds		Adults	
	Central	Peripheral	Central	Peripheral
Hits	6.77 (3.31)	4.46 (2.79)	9.52 (3.86)	6.87 (2.08)
CL	8.19 (4.70)	6.79 (4.10)	7.12 (5.86)	8.80 (3.78)
Related	2.83 (5.10)	−.48 (3.33)	3.89 (4.73)	1.67 (3.86)
*Note.* CL = critical lures.				

**Table 16 tbl16:** Means and Standard Deviations (in Parentheses) of C Values Hits, and False Alarms to Critical Lures, and Nonpresented Related Items as a Function of Age (Experiment 3)

	7/8-year-olds		Adults	
	Central	Peripheral	Central	Peripheral
Hits	.92 (3.56)	−1.36 (2.79)	.36 (3.11)	−2.29 (1.16)
CL	2.08 (5.52)	.98 (4.34)	−2.05 (5.29)	−.36 (4.02)
Related	−2.97 (4.78)	−5.96 (4.82)	−5.02 (5.03)	−7.49 (3.58)
*Note.* CL = critical lures.				

**Table 17 tbl17:** Means and Standard Deviations (in Parentheses) of Raw DRM Hits, and False Alarms to Critical Lures, and Nonpresented (Un)Related Items as a Function of Age (Experiment 3)

	7/8-year-olds	Adults
Hits	.71 (.16)	.81 (.13)
CL	.66 (.20)	.81 (.19)
Related	.18 (.16)	.21 (.20)
Unrel	.16 (.11)	.07 (.05)
*Note.* DRM = Deese/Roediger-McDermott; CL = critical lures; Unrel = unrelated.		

**Table 18 tbl18:** Means and Standard Deviations (in Parentheses) of d′ Values for DRM Hits, and False Alarms to Critical Lures, and Nonpresented Related Items as a Function of Age (Experiment 3)

	7/8-year-olds	Adults
Hits	2.28 (1.99)	4.42 (2.94)
CL	2.13 (2.42)	5.69 (4.04)
Related	−1.36 (3.02)	.43 (3.90)
*Note.* DRM = Deese/Roediger-McDermott; CL = critical lures.		

**Table 19 tbl19:** Means and Standard Deviations (in Parentheses) of C Values for DRM Hits, and False Alarms to Critical Lures, and Nonpresented Related Items as a Function of Age (Experiment 3)

	7/8-year-olds	Adults
Hits	−.33 (1.42)	.19 (2.63)
CL	−.18 (2.13)	1.47 (3.41)
Related	−3.34 (3.84)	−3.79 (3.32)
*Note.* DRM = Deese/Roediger-McDermott; CL = critical lures.		

**Table 20 tbl20:** Means and Standard Deviations (in Parentheses) of Raw Hits, and False Alarms to Critical Lures, and Nonpresented (Un)Related Items as a Function of Age (Experiment 4)

	4/5-year-olds		7/8-year-olds		11/12-year-olds	
	Central	Peripheral	Central	Peripheral	Central	Peripheral
Hits	.67 (.24)	.48 (.29)	.87 (.12)	.64 (.16)	.88 (.11)	.80 (.14)
CL	.66 (.40)	.53 (.39)	.88 (.22)	.70 (.23)	.73 (.36)	.82 (.22)
Related	.47 (.28)	.33 (.37)	.43 (.17)	.26 (.22)	.37 (.22)	.17 (.17)
Unrel	.23 (.29)		.04 (.06)		.04 (.05)	
*Note.* CL = critical lures; Unrel = unrelated.						

**Table 21 tbl21:** Means and Standard Deviations (in Parentheses) of d′ Values for Hits, and False Alarms to Critical Lures, and Nonpresented Related Items as a Function of Age (Experiment 4)

	4/5-year-olds		7/8-year-olds		11/12-year-olds	
	Central	Peripheral	Central	Peripheral	Central	Peripheral
Hits	3.41 (3.17)	2.02 (2.76)	7.18 (4.11)	4.77 (2.86)	7.31 (3.72)	6.17 (3.41)
CL	2.83 (3.33)	1.82 (2.41)	5.44 (1.74)	3.81 (1.81)	4.31 (2.76)	4.76 (1.90)
Related	1.97 (4.20)	.14 (3.92)	3.46 (4.08)	1.70 (2.92)	2.53 (4.11)	.65 (3.96)
*Note.* CL = critical lures.						

**Table 22 tbl22:** Means and Standard Deviations (in Parentheses) of C Values for Hits, and False Alarms to Critical Lures, and Nonpresented Related Items as a Function of Age (Experiment 4)

	4/5-year-olds		7/8-year-olds		11/12-year-olds	
	Central	Peripheral	Central	Peripheral	Central	Peripheral
Hits	.05 (3.87)	−1.30 (4.09)	.60 (3.11)	−1.81 (1.51)	.51 (3.45)	−.31 (3.01)
CL	.76 (6.05)	−1.17 (6.40)	3.12 (3.70)	−.14 (3.96)	1.01 (5.57)	1.72 (4.27)
Related	−1.51 (5.04)	−3.32 (6.50)	−3.12 (2.52)	−4.88 (4.25)	−3.95 (3.44)	−5.83 (3.83)
*Note.* CL = critical lures.						

**Table 23 tbl23:** Means and Standard Deviations (in Parentheses) of Raw DRM Hits, and False Alarms to Critical Lures, and Nonpresented (Un)Related Items as a Function of Age (Experiment 4)

	4/5-year-olds	7/8-year-olds	11/12-year-olds
Hits	.68 (.24)	.74 (.13)	.78 (.14)
CL	.64 (.28)	.65 (.21)	.75 (.24)
Related	.54 (.35)	.23 (.19)	.25 (.15)
Unrel	.48 (.33)	.16 (.12)	.13 (.09)
*Note.* DRM = Deese/Roediger-McDermott; CL = critical lures; Unrel = unrelated.			

**Table 24 tbl24:** Means and Standard Deviations (in Parentheses) of d′ Values DRM Hits, and False Alarms to Critical Lures, and Nonpresented Related Items as a Function of Age (Experiment 4)

	4/5-year-olds	7/8-year-olds	11/12-year-olds
Hits	.63 (1.86)	2.26 (1.88)	3.20 (2.47)
CL	.81 (2.90)	2.73 (2.68)	4.55 (3.40)
Related	−.26 (3.12)	−.80 (2.74)	.85 (2.91)
*Note.* DRM = Deese/Roediger-McDermott; CL = critical lures.			

**Table 25 tbl25:** Means and Standard Deviations (in Parentheses) of C Values DRM Hits, and False Alarms to Critical Lures, and Nonpresented Related Items as a Function of Age (Experiment 4)

	4/5-year-olds	7/8-year-olds	11/12-year-olds
Hits	1.48 (3.51)	−.08 (1.01)	−.34 (1.44)
CL	1.70 (4.37)	.39 (2.68)	1.06 (3.94)
Related	.67 (5.37)	−3.14 (3.48)	−2.64 (2.86)
*Note.* DRM = Deese/Roediger-McDermott; CL = critical lures.			

## Footnotes

1 The studies included in this review were identified using four search engines (Web of Science, PubMed, ScienceDirect, and Google Scholar). We looked for experimental papers that were written in English and published after 1978. We chose this time frame because Ceci and Bruck (1993) reported that only very few methodologically solid articles had been published before 1978. The terms we used for the literature search were: (children OR development) AND (suggestibility, misinformation, OR false memories). We also had several inclusion/exclusion criteria. We only included experiments that introduced misinformation (e.g., via suggestive questions, visual, auditory, embedded narratives) following some sort of witnessed event (classical misinformation paradigm). We did not include articles using the false memory implantation paradigm (e.g., Otgaar et al., 2010). Although these studies also introduce misinformation to participants, they follow a two-stage approach (misinformation and memory test) instead of the classic three-stage misinformation procedure (event, misinformation, and memory test) that we used in the current experiments. Also, we looked for articles that compared a minimum of two different age groups with at least one of them being a child group.

2 When we analyzed the data using the raw scores or used another transformation (two-high threshold), our developmental reversal effects in all experiments were still robust. Also, when transforming scores using *d*′ and *c*, an additional correction should be applied for values that are zero or one as the formulae for getting *d*′ and *c* values do not accept zero or one values. As there is some controversy about which correction should be used, we used the rule that the values should be adjusted slightly up (.0001) for scores of zero or slightly below for scores of one (.9999; see Snodgrass & Corwin, 1988; Stanislaw & Todorov, 1999). Of importance, using another correction (e.g., .01) did not change our main developmental trends in suggestion-based false memories. As DRM studies lead to robust levels of true and false memory, our idea was that values of 1 or 0 represent a pure signal of hit rates or rejections. Because the raw zero and one values have first been corrected and then used to calculate *d*′ and *c* values, the raw means in the tables cannot be used to accurately get the *d*′ and *c* values in the other tables.
